# Alzheimer's Aβ assembly binds sodium pump and blocks endothelial NOS activity via ROS-PKC pathway in brain vascular endothelial cells

**DOI:** 10.1016/j.isci.2021.102936

**Published:** 2021-08-04

**Authors:** Tomoya Sasahara, Kaori Satomura, Mari Tada, Akiyoshi Kakita, Minako Hoshi

**Affiliations:** 1 Department for Brain and Neurodegenerative Disease Research, Institute of Biomedical Research and Innovation, Foundation for Biomedical Research and Innovation at Kobe, CLIK 6F 6-3-7 Minatojima-Minamimachi, Chuo-ku, Kobe, 650-0047, Japan; 2 TAO Health Life Pharma Co., Ltd., Med-Pharma Collaboration Bldg, Kyoto University, 46-29 Yoshida Shimoadachi-cho, Sakyo-ku, Kyoto, 606-8501, Japan; 3 Department of Pathology, Brain Research Institute, Niigata University, Niigata, 951-8585, Japan; 4 Department of Anatomy and Developmental Biology, Graduate School of Medicine, Kyoto University, Kyoto, 606-8501, Japan

**Keywords:** Neuroscience, Clinical neuroscience, Cellular neuroscience, Cell biology

## Abstract

Amyloid β-protein (Aβ) may contribute to worsening of Alzheimer’s disease (AD) through vascular dysfunction, but the molecular mechanism involved is unknown. Using *ex vivo* blood vessels and primary endothelial cells from human brain microvessels, we show that patient-derived Aβ assemblies, termed amylospheroids (ASPD), exist on the microvascular surface in patients’ brains and inhibit vasorelaxation through binding to the α3 subunit of sodium, potassium-ATPase (NAKα3) in caveolae on endothelial cells. Interestingly, NAKα3 is also the toxic target of ASPD in neurons. ASPD-NAKα3 interaction elicits neurodegeneration through calcium overload in neurons, while the same interaction suppresses vasorelaxation by increasing the inactive form of endothelial nitric oxide synthase (eNOS) in endothelial cells via mitochondrial ROS and protein kinase C, independently of the physiological relaxation system. Thus, ASPD may contribute to both neuronal and vascular pathologies through binding to NAKα3. Therefore, blocking the ASPD-NAKα3 interaction may be a useful target for AD therapy.

## Highlights

• Unlike insoluble Aβ, ASPD preferentially exist on the endothelium of AD microvessels
• ASPD inhibit vasorelaxation via binding to ATPase α3 present on endothelial caveolae
• ASPD-NAKα3 interaction reduces NO release by increasing eNOS-Thr^495^ phosphorylation
• Unlike Aβ_1-42_, ASPD induce eNOS inactivation via mitochondrial ROS/PKC pathway

## Graphical abstract

**Figure:**
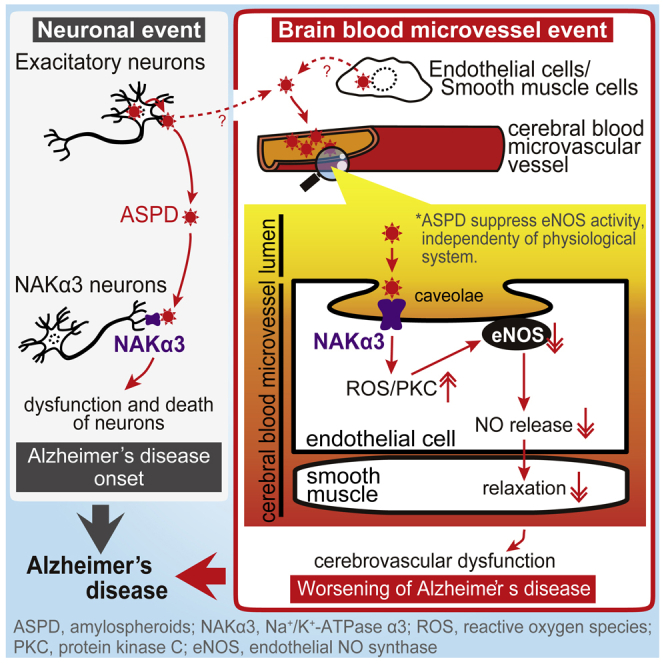

## Introduction

Alzheimer’s disease (AD) is characterized by progressive loss of neurons, deposition of aggregated forms of amyloid-β proteins (Aβs), and intracellular formation of neurofibrillary tangles (NFTs). In addition to these neuropathological features, 60-90% of the brain in AD patients exhibit vascular changes such as deposition of Aβ at cerebrovascular vessels (called cerebral amyloid angiopathy (CAA)), leading to a reduction of cerebral blood flow (Binnewijzend et al., 2016, O'brien et al., 1992), dysfunction of the blood-brain barrier (Yamazaki and Kanekiyo, 2017), induction of vascular inflammation (Suo et al., 1998), and disturbance of angiogenesis (Fischer et al., 1990), which may precede the onset of the neuropathological changes and cognitive symptoms (Govindpani et al., 2019). Recently, the symptomatic overlap between AD and vascular dementia has been focused, and the vascular biomarkers are expected to improve the clinical diagnosis of AD (Jack et al., 2018). Notably, earlier works from the Nun studies have suggested that symptomatic progression of AD related to Aβ deposition, but not to NFTs, appeared to be significantly modified by the presence of cerebrovascular abnormalities in AD (Snowdon et al., 1997). Therefore, a better understanding of the molecular mechanisms underlying Aβ-related cerebrovascular dysfunction in AD should help us to understand how vascular dysfunction contributes to AD progression and will open up new therapy.

In studies of the mechanisms of neurodegeneration in AD brains, we purified highly neurotoxic ∼30-mer assemblies of Aβ (later termed “amylospheroids”(ASPD)) from human AD brains (Hoshi et al., 2003; Noguchi et al., 2009). We proved that ASPD bind directly to the neuronal isoform of α3 subunit of the sodium pump (sodium, potassium-ATPase α3 (NAKα3)) by surface plasmon resonance analyses (*K*_d_ = 28.6 ± 6.6 nM, n = 5) and coimmunoprecipitation studies, and cause the death of mature neurons by impairing the pump activity (Ohnishi et al., 2015). ASPD levels in patients’ brains correlate well with disease severity (Ohnishi et al., 2015). Furthermore, ASPD and NAKα3 levels appeared to be inversely correlated in affected brain regions (Ohnishi et al., 2015). Interestingly, an ASPD-binding peptide, which mimics the ASPD-binding region in NAKα3, blocked ASPD neurotoxicity (Ohnishi et al., 2015). This result opens a new possibility for knowledge-based design of peptidomimetics that block the aberrant ASPD-NAKα3 interaction and thereby inhibit neurodegeneration in AD. Surprisingly, NAKα3 was also later reported to serve as a toxic target of misfolded protein assemblies, such as α-synucleins, superoxide dismutase 1 (SOD1), and tau, leading to other neurodegenerative diseases such as Parkinson'’s disease and amyotrophic lateral sclerosis (ALS) (Ruegsegger et al., 2016; Shrivastava et al., 2015, 2019). This illustrates the value and generality of NAKα3 impairment in neurodegeneration.

Recently, we established a mature neuron-based system that allows us to chronologically follow ASPD formation in mature neurons (Komura et al., 2019). With this system, we found that ASPD accumulate mainly in the *trans*-Golgi network of excitatory neurons and are secreted through as-yet-unknown mechanisms, leading to the death of adjacent NAKα3-expressing neurons (Komura et al., 2019). This finding led us to explore the possibility that secreted ASPD may reach the blood vessels and contribute to the cerebrovascular changes in AD brains. Here, by using *in vitro* blood cell cultures and *ex vivo* blood vessels, we showed that ASPD bind to NAKα3 in endothelial cells, as we had previously found in neurons (Ohnishi et al., 2015), and inhibit the pump function. But, in contrast to mature neurons, the aberrant ASPD-NAKα3 interaction in endothelial cells induces production of ROS in mitochondria and activates protein kinase C (PKC). This increases the PKC-phosphorylated inactive form of endothelial nitric oxide (eNOS), and decreases nitric oxide (NO) production. This in turn would suppress the relaxation of blood microvessels and might cause a reduction of cerebral blood flow and other vascular dysfunctions in AD brains. Thus, we show a new possibility that brain Aβ assemblies accelerate worsening AD pathologies by affecting the cerebrovascular systems via interaction with the sodium pump.

## Results

### ASPD are present in cerebrovascular vessels of AD brain

**Figure 1:**
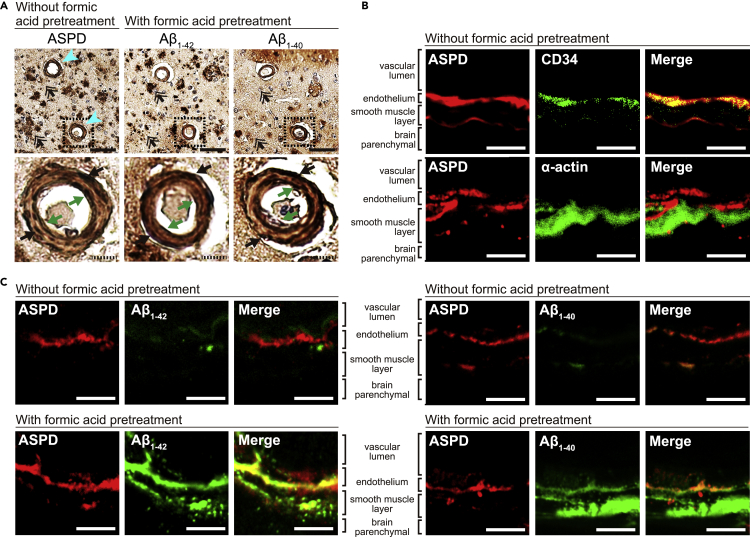
ASPD are present in cerebrovascular vessels of AD brain (A) Immunohistochemical staining of serial sections of frontal cortex of AD patients, using antibodies specific for ASPD (rabbit polyclonal rpASD1 antibody (Noguchi et al., 2009)) (left), Aβ_1-42_ (middle), and Aβ_1-40_ (right) (see “method details”). While the sections were pretreated with formic acid to detect insoluble Aβ with Aβ_1-42_ or Aβ_1-40_ antibody, no such pretreatment was used to detect soluble ASPD (see “method details”). Representative images (upper panels) and enlarged views of the area surrounded by the hatched line (lower panels) are shown. In the upper panels, turquois arrowheads on the left indicate microvessels stained by rpASD1 antibody, and double-lined arrows indicate ASPD staining that seemingly does not overlap with the insoluble Aβ_1-42_ or Aβ_1-40_ plaques. In the lower panels, green arrows indicate each antibody’s staining in the endothelial layer, while black arrows mark that in the smooth muscle layer. Scale bars: 100 μm for solid line and 10 μm for hatched line. (B) Double immunofluorescence staining of serial sections of the frontal cortex of the same AD patients in A was performed with ASPD-specific antibody (rabbit polyclonal rpASD1 in the upper panels, mouse monoclonal mASD3 in the lower panels), along with antibodies for blood vessel endothelial CD34 or smooth muscle α-actin (see “method details”), without tissue pretreatment. Scale bars: 5 μm. (C) Double immunofluorescence staining of serial sections of the frontal cortex of the same AD patients in A was performed, with or without formic acid pretreatment, with ASPD-specific antibody (mouse monoclonal mASD3), along with antibodies for Aβ_1-42_ and Aβ_1-40_ (see “method details”). Enhanced fluorescence images of Aβ staining on the section without pretreatment are shown in FigureS1. Scale bars: 5 μm.

We first examined whether ASPD accumulate in blood microvessels of the frontal cortex of three AD patients’ brains (their profiles are shown in Table 1), using in-house-established ASPD-tertiary-structure-dependent antibodies (rabbit polyclonal rpASD1 and mouse monoclonal mASD3), which selectively detect ASPD in cell/tissue staining and show little cross-reactivity with other Aβ oligomers recognized by a pan-Aβ oligomer A11 antibody (see Table S1 in (Noguchi et al., 2009) for summary). Because the naïve ASPD tertiary structure is best detected in tissue sections without pretreatment, such as formic acid (Noguchi et al., 2009), ASPD staining was obtained without any pretreatment. A representative staining in Figure 1A upper left shows that ASPD are widely accumulated around senile plaques and neurons (as reported in (Noguchi et al., 2009)). In addition to this brain parenchymal staining, we also detected ASPD in most microvessels (turquoise arrowheads in Figure 1A upper left). From the expanded view in Figure 1A lower left, ASPD appeared to accumulate in the endothelial layer on the inner surface of the microvessels (green arrows) as well as the smooth muscle layer outside (black arrows). Unlike ASPD, insoluble Aβ is barely detectable without formic acid pretreatment (Christensen et al., 2009; Noguchi et al., 2009). Accordingly, insoluble Aβs were stained with antibodies for Aβ_1-42_ and Aβ_1-40_ using the tissues pretreated with formic acid. As shown in Figure 1A, insoluble Aβ staining, particularly Aβ_1-42_, overlapped with ASPD staining, but does not match completely (compare double-lined arrows among upper panels in Figure 1A).

**Table 1:** AD profiles of the three patients whose brains were utilized in this study

	AD patients		
	1	2	3
Age (year)	86	87	79
Sex	Female	Female	Male
Clinical diagnosis	AD	AD	CBS
Disease duration (year)	21	9	4
NFT (Braak stage)	VI	V	V
SP (Braak stage)	C	C	C
Amyloid angiopathy	+	+	+
Postmortem delay (hr)	4	3	8
Brain weight (g)	995	1,000	1,000

AD, Alzheimer's disease; CBS, corticobasal syndrome; NFT, neurofibrillary tangle; SP, senile plaque.

We next examined a more precise location of ASPD in the microvessels of the above patients’ brains by using double immunofluorescence staining. The results in Figure 1B show that ASPD co-localize almost completely with CD34, a marker of the blood vessel endothelium (Sidney et al., 2014), but only partially with smooth muscle α-actin, suggesting that ASPD are present more abundantly in the endothelium. As a control, co-immunostaining of ASPD with Aβ_1-42_ or Aβ_1-40_ was performed without formic acid pretreatment. As expected, Aβ staining was very weak (Figure 1C upper panels). Nevertheless, the enhanced fluorescence intensity of Aβ revealed very limited overlap between Aβ and ASPD signals in the endothelial layer (see white arrowheads and the line-scan analysis in FigureS1). This result is reasonable, because our previous mass analyses have shown that ASPD purified from AD patients’ brains are composed of both Aβ_1-42_ and Aβ_1-40_ (Noguchi et al., 2009). When the serial sections of the same patients’ brains were pretreated with formic acid, substantial accumulations of Aβ_1-42_ and Aβ_1-40_ were observed in both the endothelial layer and the smooth muscle layer (Figure 1C lower panels), as reported previously (Keable et al., 2016). In contrast, ASPD were mainly detected in the endothelial layer (Figure 1C lower panels). These results collectively support our previous conclusion that soluble ASPD is present independently, albeit seemingly partially overlapping with insoluble Aβ accumulation (Noguchi et al., 2009). Thus, ASPD are present both in the parenchyma and the microvessels, preferentially in the endothelial layer, of human AD patients’ brains. In contrast, insoluble Aβ accumulates both in the endothelial and the smooth muscle layers. Accordingly, we focused on the effect of ASPD on brain endothelial cells in this work.

### ASPD inhibit relaxation of blood vessels through binding to endothelial NAKα3

**Figure 2:**
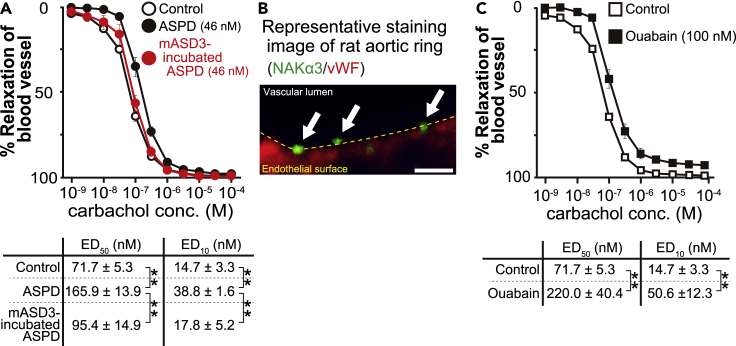
ASPD inhibit *ex vivo* relaxation response of blood vessels through NAKα3 inhibition (A–C) The effect of ASPD (with or without 2-hr preincubation with ASPD-specific mASD3 antibody) in A or ouabain in C on the carbachol dose-dependent induction of the relaxation response of phenylephrine-constricted *ex vivo* rat aortic rings. The rat isolated aortic rings were treated with ASPD, ASPD preincubated with mASD3 antibody (0.1 mg/mL) (Noguchi et al., 2009; Ohnishi et al., 2015), or ouabain (an inhibitor for rodent NAKα3 at the concentration used) at the indicated concentrations and the carbachol-induced relaxation response was examined by monitoring the isometric tension change (see “method details”). Data are expressed as a percentage to the maximal constriction induced by phenylephrine (n = 5, except for mASD3-preincubated ASPD (n = 3)). *In vitro*-reconstituted synthetic ASPD, which share essential characteristics with patient-derived ASPD (see Table S1 in (Ohnishi et al., 2015)), were used generally, except for the experiments in Figure 7. ED_50_ and ED_10_ values ofcarbachol required for relaxation are shown below the plots. Data are presented as means ± S.E. ∗∗*P* < 0.01 (ANOVA with Scheffé’s method (A) and Welch’s *t* test (C)). (B) Double immunofluorescence staining of rat aortic rings prepared as in A was performed with antibodies specific for NAKα3 and vWF (see “method details”). The arrows indicate NAKα3 on the apical surface of the endothelium. Scale bars: 1 μm.

Endothelial cells produce and release three main types of vascular relaxation factors–NO, prostacyclin, and endothelium-derived hyperpolarizing factor (EDHF)–leading to relaxation of blood vessel smooth muscles (Arnold et al., 1977; Furchgott and Zawadzki, 1980; Giles et al., 2012; Ignarro et al., 1987). Among these relaxation factors, NO plays a major role in the vascular relaxation reaction in large blood vessels, while EDHF plays a more important role in microvessels (Giles et al., 2012). However, recent studies have shown that eNOS plays a major role in producing not only NO in large blood vessels but also H_2_O_2_, a major EDHF, in microvessels (Shimokawa and Godo, 2016). Therefore, we examined whether ASPD affect the relaxation response of blood vessels through altering eNOS activity. Due to the limited availability of human blood microvessels that are sufficiently fresh for functional studies, we tested the effect of ASPD on aortic rings isolated from rats, because the aortic ring is the most sensitive vascular system that can detect changes in the eNOS-dependent relaxation response (see review by (Shimokawa and Godo, 2016)), and the overall reaction of rodent blood vessels is quite similar to that of human blood vessels, e.g., in magnitude of maximum response, sensitivity, and molecular mechanisms of agonists, including carbachol, which we used in this work (Fuchikami et al., 2017; Grande et al., 2013; Karpinska et al., 2017; Osol et al., 2008; Sheykhzade et al., 2018). The aortic rings, isolated according to the established method (Angus and Wright, 2000; Sasahara et al., 2013), were contracted by treatment with phenylephrine (an adrenergic α1 receptor agonist), and NO-dependent relaxation was induced by carbachol (a muscarinic receptor agonist). On mature neurons, ASPD affect in a dose-dependent manner, and the effect reaches a plateau at ∼40 nM (Ohnishi et al., 2015) (Note that ASPD concentrations are shown based on the average mass of ASPD, 128 kDa.). We, therefore, used this plateau concentration of ASPD for the present experiments. Treatment of the isolated aortic rings with 46 nM ASPD for 1 hr inhibited the carbachol-induced relaxation response (Figure 2A upper) and doubled the ED_50_ values of carbachol required for relaxation (Figure 2A lower). We also present the ED_10_ values because the change of blood vessels in the actual brain generally takes place within a narrow range as it is directly linked to the blood pressure. As shown in Figure 2A, ASPD also doubled the ED_10_. When ASPD were preincubated with mASD3 antibody that blocks ASPD binding to neurons (Noguchi et al., 2009; Ohnishi et al., 2015), the increase in ED_50_ and ED_10_ was completely abolished (Figure2A; *p* values of the ED_50_ and ED_10_ between the untreated control and the mASD3-preincubated ASPD were 0.52 and 0.50, respectively). These results show that ASPD directly suppress the NO-dependent relaxation of the blood vessels, probably through affecting eNOS in endothelial cells.

Immunostaining of AD patients (Figures 1A and 1B) revealed that ASPD were also accumulated on smooth muscles. Therefore, to rule out the possibility that ASPD act directly on the smooth muscles of blood vessels, we confirmed that ASPD did not affect the relaxation response induced by papaverine, which directly relaxes blood vessel smooth muscles in an endothelium-independent manner (Lugnier et al., 1972; Martin et al., 1986). Indeed, 46 nM ASPD did not affect either the papaverine-induced relaxation response of the aortic rings (% maximal relaxation induced by papaverine: 100.9 ± 0.5 and 101.4 ± 0.7% with and without ASPD, respectively; n = 5, *P* = 0.46) or the time to reach the maximal relaxation (3.4 ± 0.1 and 3.6 ± 0.3 min with and without ASPD, respectively; n = 5, *P* = 0.69). These results collectively support the idea that ASPD acted on endothelial cells in the above *ex vivo* experiments (see also discussion in “Limitations of the Study” section).

Next, we set out to identify the target protein on endothelial cells to which ASPD bind to inhibit NO release. We previously found that ASPD impair neuron-specific sodium pump activity by binding to the NAKα3 subunit in neurons, leading to neurodegeneration (Ohnishi et al., 2015). We therefore speculated that NAKα3 might also serve as an ASPD toxic target in endothelial cells and mature neurons (Ohnishi et al., 2015). Because NAKα3 is a neuron-specific isoform (Shrivastava et al., 2020), we first examined whether NAKα3 is present on the endothelial cell surface by immunostaining. We detected patchy NAKα3 staining (green signals indicated by arrows in Figure 2B) on the vascular lumen surface of the endothelial cells of the isolated aortic rings (red shows a signal of von Willebrand factor (vWF) glycoprotein, an endothelial cytoplasmic marker (Rakocevic et al., 2017)). To confirm the functional involvement of NAKα3 in the suppression of the blood relaxation response, we examined the effect of 100 nM ouabain, a concentration that is enough to inhibit the rodent NAKα3 isoform, but not other rodent NAKα isoforms (Noel et al., 1990). As shown in Figure 2C, this concentration of ouabain sufficiently inhibited the relaxation response and increased both the ED_50_ and the ED_10_ of carbachol required for relaxation of the blood vessels, as observed in the ASPD treatment (Figure 2A). These results collectively support the idea that ASPD suppress blood vessel relaxation by affecting endothelial cell function through inhibition of NAKα3 pump activity. Because the relaxation response of microvessels plays a key role in blood pressure regulation, we decided to use primary cultures of endothelial cells obtained from human brain microvessels to further dissect the molecular action of ASPD.

### ASPD suppress NO release by binding to NAKα3 in primary human cerebral endothelial cells

**Figure 3:**
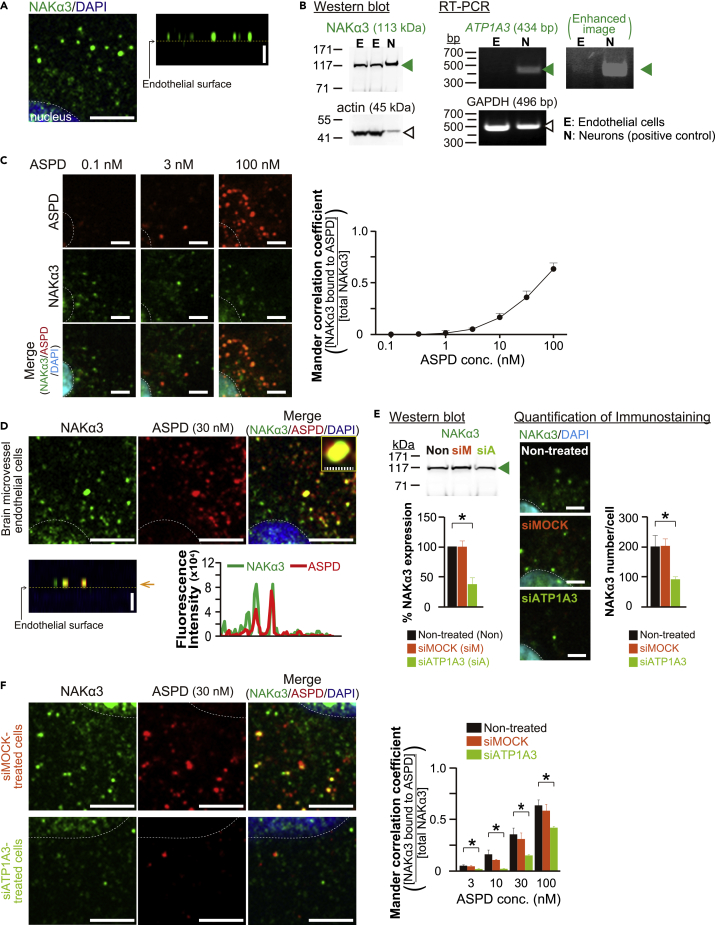
Binding target of ASPD on human brain microvessel endothelial cells is NAKα3 (A) Primary human brain microvessel endothelial cells were stained with NAKα3-specific antibody and DAPI nuclear stain, and high-power fluorescence images were acquired using a confocal laser-scanning microscope LSM710 with a x100 oil-immersion objective lens (see “method details”). Representative 2D images are shown on the left and the vertical section image obtained from the ZStack 3D images is shown on the right. Scale bars: 5 μm. (B) NAKα3 expression in the cells in A was determined by Western blotting using a NAKα3-specific antibody (left panels) or by RT-PCR analysis of *ATP1A3* mRNA (right panels) (see “method details”). For Western blotting, 70 μg and 5 μg proteins were loaded for endothelial cells and primary neurons, respectively (see “method details”). (C) Endothelial cells treated with ASPD at the indicated concentration for 10 min were fixed and stained with ASPD-specific mASD3 antibody (Noguchi et al., 2009), NAKα3-specific antibody, and DAPI nuclear stain (see “method details”). The fluorescence 2D images were captured using a confocal quantitative image cytometer CQ1 (left panels), and the ratio of ASPD-bound NAKα3 to total NAKα3 was obtained as a Mander correlation coefficient using CQ1 software (right, n = 5) (see “method details”). Scale bars: 5 μm. Data are presented as means ± S.E. (D) High-power 2D fluorescence images of the cells treated with ASPD (30 nM) in C were acquired as in A (upper). The vertical section image on the lower left and the line-scan analysis of the fluorescence intensity of NAKα3 (green line) or ASPD (red line) on the lower right were obtained from the ZStack 3D images of the same cells using Zen2009 software (see “method details”). Scale bars: 5 μm for solid line and 1 μm for hatched line. (E) The expression levels of NAKα3 in the cells without transfection or with *ATP1A3* siRNA (s1724, Thermo Fisher Scientific) or MOCK siRNA (negative control, 4390843, Thermo Fisher Scientific) transfection for 3 days were determined by Western blotting (left) and by quantification of NAKα3 staining (right) (see “method details”). Data in the lower left are shown as the ratio of NAKα3 to actin, and the ratio for the non-treated cells is shown as 100 (n = 3) (see “method details”). 2D immunofluorescence images of NAKα3 staining as shown on the left were captured as in C. The NAKα3 number/cell on the right was calculated by dividing the number of punctate NAKα3 stains by the number of DAPI stains using CQ1 software (n = 5) (see “method details”). Scale bars: 5 μm. Data are presented as means ± S.E. ∗*P* < 0.05 (ANOVA with Scheffé’s method). (F) Endothelial cells, without transfection, or with *ATP1A3* siRNA or MOCK siRNA transfection as in E, were treated with ASPD (30 nM) for 10 min, and were stained with specific antibodies as in C. High-power 2D fluorescence images of the cells were obtained as in A (left panels). The ratio of the ASPD-bound NAKα3 to total NAKα3 was obtained as a Mander correlation coefficient, as in C (right, n = 5). Scale bars: 5 μm. Data are presented as means ± S.E. ∗*P* < 0.05 (ANOVA with Scheffé’s method).

We first confirmed NAKα3 expression in human brain microvessel-derived endothelial cells. Immunostaining detected punctate NAKα3 signals scattered on the cell surface (Figure 3A; representative 2D image on the left and the vertical views of the ZStack images on the right). Western blotting showed the presence of NAKα3 in the endothelial cells (green arrowhead in Figure 3B left). RT-PCR analysis further confirmed NAKα3 expression (green arrowhead in Figure 3B right). All these results indicated the presence of NAKα3 in human brain microvessel-derived endothelial cells.

We next examined whether ASPD interact with the endothelial NAKα3 using immunostaining. As shown in Figure 3C left panels, binding of ASPD to the endothelial NAKα3 increased dose-dependently (see quantification in Figure 3C right). Quantification showed that the ratio of the ASPD-bound NAKα3 to total NAKα3 increased according to the ASPD concentration and reached 63.4 ± 5.7% at 100 nM ASPD (n = 5, Figure 3C right). A high-power image showed that the ASPD and NAKα3 signals are essentially overlapped (Figure 3D upper panels and inset). The vertically sectioned image and its line scan (Figure 3D lower panel) indicated that the interaction of ASPD and NAKα3 takes place on the endothelial cell surface.

To further confirm ASPD-NAKα3 interaction on the brain endothelial cells, we examined whether knockdown of NAKα3 expression by small interfering RNA (siRNA) blocks the interaction of ASPD and NAKα3. Western blotting and immunostaining consistently showed that the transfection of *ATP1A3* siRNA decreased the NAKα3 level to 38 ± 11% (n = 3) and 45 ± 5% (n = 5) of the level in the non-transfected cells, respectively (Figure 3E). In parallel with the decrease of NAKα3 level, the *ATP1A3* siRNA transfection decreased the ratio of the ASPD-bound NAKα3 to total NAKα3 to 31 ± 11% of that in the non-treated cells on average (n = 5, Figure 3F; compare the images among the non-transfected, mock-transfected, and *ATP1A3*-transfected cells in Figures 3D and 3F). Mock siRNA transfection did neither affect the NAKα3 expression levels (Figure 3E) nor the ratio of the ASPD-bound NAKα3 to total NAKα3 (Figure 3F). The siRNA interference results support the conclusion that ASPD interact with NAKα3 on the brain endothelial surface.

**Figure 4:**
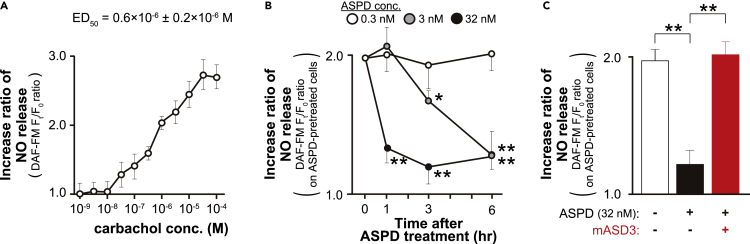
ASPD suppress NO release from human brain microvessel endothelial cells (A) NO release was determined using an NO fluorescence indicator, DAF-FM. The cells were loaded with DAF-FM (see “method details”), and further treated with carbachol for 5 min at the indicated concentrations. DAF-FM-derived fluorescence intensities, before and after the carbachol treatment, measured using a laser scanning cytometer CQ1, were defined as F_0_ and F_t_, respectively (see “method details”). Data are shown as the ratio of F_t_ to F_0_ (n = 5). A semi-log scale was utilized to obtain ED_50_ of carbachol action on NO release (ED_50_ = 0.6 ± 0.2 μM; n = 5). As a negative control, we confirmed that pretreatment with NOS inhibitor *L*-NAME abolished the increase of fluorescence intensity induced by carbachol (see “method details”). Data are presented as means ± S.E. (B) The cells were pretreated with ASPD (0.3, 3, or 32 nM) for 0, 1, 3, or 6 hr (see “method details”), and the change of DAF-FM-derived fluorescence intensity by carbachol (1 μM) was measured as in A (n = 4). Data are presented as means ± S.E. ∗*P* < 0.05/∗∗*P* < 0.01 (ANOVA with Scheffé’s method). (C) NO release was compared among the non-treated, ASPD-treated, and mASD3-preincubated ASPD (prepared as in Figure 2A)-treated cells. The increase ratio of NO release was obtained as in A (n = 4). Data are presented as means ± S.E. ∗∗*P* < 0.01 (ANOVA with Scheffé’s method).

To elucidate the effect of ASPD on eNOS activity in these human brain endothelial cells, we next determined the ED_50_ of carbachol required for NO release using diaminofluorescein-FM (DAF-FM; Sekisui Medical, Tokyo, Japan), a fluorescent probe for NO quantification (Kojima et al., 1999). Treatment of the cells with carbachol for 5 min increased NO release dose-dependently, which reached a plateau at around 100 μM (Figure 4A). For further experiments, we treated primary human brain microvessel endothelial cells with carbachol at 1 μM, which was approximately the ED_50_ concentration (0.6 ± 0.2 μM, n = 5) required for NO release, estimated in Figure 4A.

We found that treatment of the primary human brain microvessel endothelial cells with ASPD antagonized the observed carbachol-induced NO release in a dose- and time-dependent manner (Figure 4B). This means that the NO release decreased more rapidly and more strongly in correlation with the increase in the ASPD binding ratio to the endothelial NAKα3 (compare Figure 3C right with Figure 4B). For example, 32 nM ASPD, which interacted with 35 ± 6% of total NAKα3 (n = 4, Figure 3C right), fully inhibited the carbachol-induced NO release after 3 hr incubation (22 ± 11%, n = 4), while 3 nM ASPD, which interacted with 5.2 ± 0.9% of total NAKα3 (n = 4, Figure 3C right), required 6 hr to reach the maximal inhibition (30 ± 18%, n = 4). In contrast, 0.3 nM ASPD, which interacted with 0.7 ± 0.2% of total NAKα3 (n = 4, Figure 3C right), had no effect on the NO release during incubation for up to 6 hr (Figure 4B). The observed antagonistic effect was attributable to ASPD, as the ASPD-specific mASD3 antibody that inhibits ASPD binding to NAKα3 (Noguchi et al., 2009; Ohnishi et al., 2015) almost completely abolished the effect (Figure 4C). Binding and functional analyses (Figures 3 and 4) together support the conclusion that ASPD antagonized carbachol-induced NO release through binding to NAKα3.

### ASPD-NAKα3 interaction in caveolae increases the phosphorylation of eNOS-Thr^495^ in primary human cerebral endothelial cells

The above findings suggest that ASPD-NAKα3 interaction inhibits the activity of eNOS. eNOS is primarily localized to plasma membrane microdomains where it binds to caveolin-1 and the Golgi apparatus. Upon stimulation, eNOS dissociates from caveolin-1 and generates NO. Thus, NO release by eNOS takes place in close proximity to caveolin-1-enriched microdomains termed caveolae in the endothelial cells (Zhang et al., 2006). Notably, NAKα1 is a caveolin-1-binding protein, and newly synthesized NAKα1 is transferred to caveolae in the plasma membrane from the Golgi apparatus (Cai et al., 2008). Because NAKα3 completely preserves the caveolin-1-binding motifs in transmembrane domains M1 and M10 (see Table 2) (Yosef et al., 2016), it is reasonable to consider that NAKα3 also resides in caveolae, along with eNOS. Therefore, we first examined the colocalization of eNOS and NAKα3.

**Table 2:** Comparison of caveolin-1-binding motifs in human NAKα1 and NAKα3

caveolin-binding motif in TM 1		φ	X	X	Φ	X	X	X	X	φ	X	X	φ	X	X	X	X	Φ			
Human NAKα1	E	W	I	K	F	C	R	Q	L	F	G	G	F	S	M	L	L	W	I	G	A
Human NAKα3	E	W	V	K	F	C	R	Q	L	F	G	G	F	S	I	L	L	W	I	G	A
Caveolin-binding motif in TM 10							φ	X	Φ	X	X	X	X	φ	X	φ					
Human NAKα1	T	W	W	F	C	A	F	P	Y	S	L	L	I	F	V	Y	D	E	V	R	K
Human NAKα3	S	W	W	F	C	A	F	P	Y	S	F	L	I	F	V	Y	D	E	I	R	K

The first line shows the caveolin-binding motifs in transmembrane (TM) 1 and 10, reported in (Yosef et al., 2016), in which φ and X mean an aromatic amino acid and any amino acid, respectively. The caveolin-1-binding motifs in human NAKα1 (UniPlot: P05023) and those preserved in human NAKα3 (UniPlot: P13637) are shown in the second and third lines, respectively.

**Figure 5:**
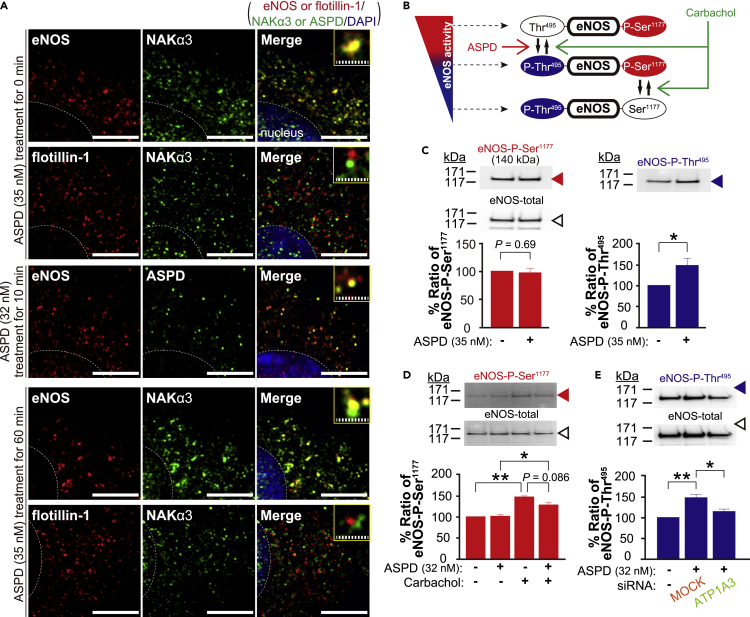
ASPD-NAKα3 interaction in caveolae increases the phosphorylation of eNOS-Thr^495^ in primary human cerebral endothelial cells (A) Primary human brain microvessel endothelial cells, treated with ASPD at the indicated concentration for 0, 10, or 60 min, were multiply stained with the indicated antibodies; NAKα3-specific antibody, eNOS-specific antibody, lipid rafts flotillin-1-specific antibody, and ASPD-specific antibody (rabbit polyclonal rpASD1), along with DAPI nuclear stain, were used as described in “method details”. The weighted colocalization coefficients were obtained using ZEN2009 software (the eNOS-overlapped NAKα3/total NAKα3 ratios in the cells treated with ASPD for 0, 10, and 60 min are 55.7 ± 2.4%, 58.8 ± 4.8%, and 56.0 ± 2.4%, respectively; the NAKα3-overlapped eNOS/total eNOS ratios in the cells treated with ASPD for 0, 10, and 60 min are 40.8 ± 1.4%, 42.7 ± 2.1%, and 40.1 ± 1.7%, respectively; the flotillin-1-overlapped NAKα3/total NAKα3 ratios in the cells treated with ASPD for 0, 10, and 60 min are 4.5 ± 0.9%, 4.9 ± 1.2%, and 3.6 ± 0.9%, respectively: data are presented as means ± S.E. (n = 10)) (see “method details”). The weighted colocalization coefficients represent the number of red (or green) pixels that co-localize with green (or red) pixels divided by the total number of red (or green) pixels. Scale bars: 5 μm for solid line and 1 μm for hatched line. (B) Schematic illustration of the relationship between the NO production and the phosphorylation at Ser^1177^/Thr^495^ of eNOS. Carbachol activates eNOS by inducing phosphorylation at Ser^1177^ and dephosphorylation at Thr^495^ in parallel (green arrows). ASPD increase Thr^495^ phosphorylation of eNOS (red arrow) through an independent pathway from that of carbachol. (C) Primary human endothelial cells were treated with ASPD (35 nM) for 6 hr (see “method details”). The levels of eNOS-P-Ser^1177^, eNOS-P-Thr^495^, and eNOS-total were determined by Western blotting of total extracts with antibodies specific for eNOS-P-Ser^1177^, eNOS-P-Thr^495^, and eNOS-total, respectively, as shown in upper Western blots (see “method details”). Quantification data were determined by densitometry using LAS-4000 Mini software and are shown as the ratio of eNOS-P-Ser^1177^ or eNOS-P-Thr^495^ to eNOS-total. The ratio of non-treated cells is shown as 100 (n = 4). Data are presented as means ± S.E. (Welch’s *t*-test). (D) The endothelial cells were pretreated with ASPD (32 nM) for 6 hr and were further treated with carbachol (1 μM) for 5 min (see “method details”). The ratio of eNOS-P-Ser^1177^ to eNOS-total was obtained and is shown as in C (n = 4). Data are presented as means ± S.E. ∗*P* < 0.05/∗∗*P* < 0.01 (ANOVA with Scheffé’s method). (E) The endothelial cells, with *ATP1A3* siRNA or Mock siRNA transfection or without transfection as in Figure 3E, were treated with ASPD (32 nM) for 6 hr. The ratio of eNOS-P-Thr^495^ to eNOS-total was obtained as in C (n = 3). Data are presented as means ± S.E. ∗*P* < 0.05/∗∗*P* < 0.01 (ANOVA with Scheffé’s method).

The immunofluorescence images in Figure 5A show that the eNOS and NAKα3 signals overlapped well before ASPD treatment. Scattering analyses of these images revealed the eNOS-overlapped NAKα3/total NAKα3 and the NAKα3-overlapped eNOS/total eNOS ratios were 55.7 ± 2.4% and 40.8 ± 1.4% (n = 10), respectively. In contrast, the flotillin-1 (a marker of the lipid microdomains (Smart et al., 1999))-overlapped NAKα3/total NAKα3 ratio was 4.5 ± 0.9% (n = 10). This result supports the idea that eNOS and NAKα3 are present in the same microdomains, caveolae. We confirmed that ASPD signals overlapped with eNOS (Figure 5A); the eNOS-overlapped ASPD/total ASPD ratio was 79.4 ± 2.9% (n = 10) after 10 min incubation of the cells with 32 nM ASPD. Interestingly, we also found that ASPD treatment, up to 60 min, the time point at which NO release was maximally decreased after ASPD treatment (Figure 4B), did not change the overlapping ratio of eNOS and NAKα3 (Figure5A); e.g., the eNOS-overlapped NAKα3/total NAKα3 and the NAKα3-overlapped eNOS/total eNOS ratios were 56.0 ± 2.4% and 40.1 ± 1.7% (n = 10), respectively, after 60 min incubation of the endothelial cells with 32 nM ASPD. At this time point, the flotillin-1-overlapped NAKα3/total NAKα3 ratio was 3.6 ± 0.9% (n = 10). The above data collectively support that ASPD-NAKα3 interaction takes place in caveolae, in close proximity to eNOS.

Past studies have shown that enzymatic eNOS activity is mainly regulated by the above shown subcellular localization (Zhang et al., 2006) and phosphorylation (Heiss and Dirsch, 2014). Because ASPD did not seem to change the topological proximity between eNOS and NAKα3, we next examined whether ASPD inhibit the activity of eNOS by changing its phosphorylation state. The eNOS activity is regulated through phosphorylation at Ser^1177^ and Thr^495^, which are regulated by distinct kinases and phosphatases, respectively (Heiss and Dirsch, 2014); phosphorylation of eNOS-Ser^1177^ (eNOS-P-Ser^1177^) activates, while phosphorylation of eNOS-Thr^495^ (eNOS-P-Thr^495^) deactivates (see scheme in Figure 5B). As for carbachol, it simultaneously activates the kinase responsible for phosphorylating eNOS-Ser^1177^ and the phosphatase responsible for dephosphorylating eNOS-Thr^495^ (see green arrows in Figure 5B). Western blotting showed that ASPD, in the absence of carbachol, increased the eNOS-P-Thr^495^ ratio without changing the eNOS-P-Ser^1177^ ratio (Figure 5C). Note that ASPD did not change the total eNOS level after 6 hr incubation (compare the blots of eNOS-total in Figure 5C). This suggests that ASPD regulate eNOS-Thr^495^ phosphorylation independently of carbachol (red arrow in Figure 5B). Consistently, ASPD did not significantly affect the eNOS-P-Ser^1177^ ratio in the presence of carbachol (Figure 5D). Finally, we confirmed that the ASPD-NAKα3 interaction was responsible for the increase in eNOS-Thr^495^ phosphorylation by knocking down NAKα3 expression with siRNA. As shown in Figure 5E, the transfection of the endothelial cells with *ATP1A3* siRNA completely blocked the ASPD-induced eNOS-P-Thr^495^. These results collectively show that ASPD-NAKα3 interaction negatively regulates the relaxation response of the blood vessels through eNOS-Thr^495^ phosphorylation independently of the usual relaxation mechanisms induced by carbachol.

### The mitochondrial ROS/PKC pathway is involved in eNOS-Thr^495^ phosphorylation by ASPD in primary human cerebral endothelial cells

**Figure 6:**
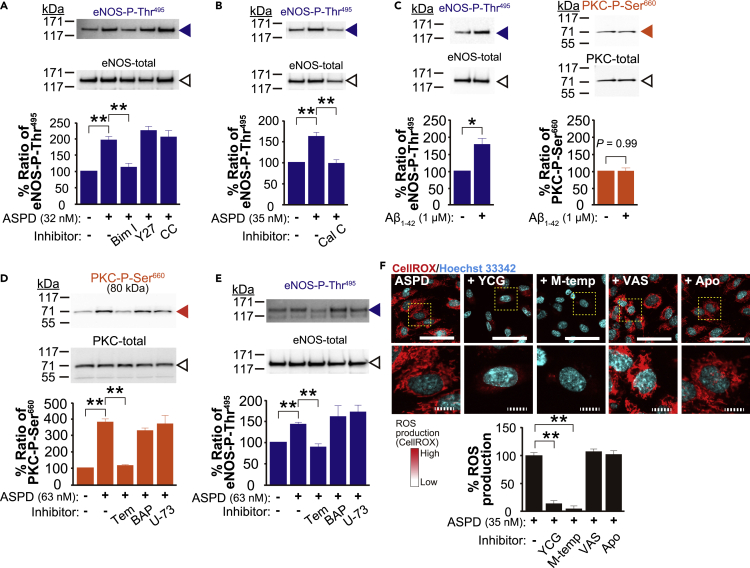
The mitochondrial ROS/PKC pathway is involved in eNOS-Thr^495^ phosphorylation by ASPD in primary human cerebral endothelial cells (A and B) Primary human endothelial cells were pretreated for 30 min either with bisindolylmaleimide I (Bim I, 5 μM), Y-27632 (Y27, 10 μM), or compound C (CC, 10 μM) in A or with calphostin C (Cal C, 0.3 μM) in B, and were further treated for 6 hr with ASPD (32 nM in A or 35 nM in B) (see “method details”). The ratio of eNOS-P-Thr^495^ to eNOS-total was obtained as in Figure 5C. The ratio in the non-treated cells is shown as 100 (n = 4 in A and 3 in B). As shown in Western blots in B, calphostin C decreased the eNOS-total without affecting cell survival, most likely due to its non-specific inhibition of phospholipase D activity (Zheng et al., 2004). Nevertheless, because both bisindolylmaleimide I and calphostin C completely inhibited the ASPD-induced increase in eNOS-P-Thr^495^, this does not affect the conclusion that ASPD work through PKC activation. Data are presented as means ± S.E. ∗∗*P* < 0.01 (ANOVA with Scheffé’s method). (C) The cells were treated with freshly dissolved Aβ_1-42_ solution (1 μM) for 6 hr (see “method details”). The ratio of eNOS-P-Thr^495^ to eNOS-total was determined as in A. The ratio of PKC-P-Ser^660^ to PKC-total was determined by Western blotting of total extracts with antibodies specific for PKC-P-Ser^660^ and PKC-total, respectively (n = 4). The results show that ASPD induce eNOS inactivation through PKC activation, whereas freshly dissolved Aβ_1-42_ does not. Our findings also confirmed a possible link between Aβ and inactivation of eNOS, the molecular mechanism of which has been largely unknown, as previously reported (Chisari et al., 2010; Gentile et al., 2004; Lamoke et al., 2015; Suhara et al., 2003). AMP kinase and Rho kinase have been also reported to play a role in the physiological regulation of eNOS by phosphorylating Thr^495^ (Heiss and Dirsch, 2014). These kinases might be involved in the case of Aβ_1-42_. Data are presented as means ± S.E. ∗*P* < 0.05 (Welch's *t*test). (D and E) The cells were pretreated with tempol (Tem, 3 mM), BAPTA-AM (BAP, 30 μM), or U-73122 (U-73, 10 μM) for 30 min, and were further treated with ASPD (63 nM) for 6 hr (see “method details”). The ratios of eNOS-P-Thr^495^ to eNOS-total and that of PKC-P-Ser^660^ to PKC-total were obtained as above (n = 4). Data are presented as means ± S.E. ∗∗*P* < 0.01 (ANOVA with Scheffé’s method). (F) The cells were pretreated with YCG-063 (YCG, 50 μM), mito-tempol (M-temp, 100 μM), VAS2870 (VAS, 10 μM), or apocynin (Apo, 20 μM) for 30 min (see “method details”), and were further treated with ASPD (35 nM) for 6 hr. ROS production was estimated by monitoring the fluorescence intensity of a ROS fluorescence indicator, CellROX (see “method details”) (n = 4). Representative fluorescence images, along with expanded images of the areas surrounded by hatched lines in the upper panels are shown. The CellROX fluorescence intensities were determined using a laser scanning cytometer CQ1 (see “method details”). Quantification data in ASPD-treated cells are shown as 100. Scale bars: 100 μm for solid line and 20 μm for hatched line. Data are presented as means ± S.E. ∗∗*P* < 0.01 (ANOVA with Scheffé’s method).

We next clarified how the ASPD-NAKα3 interaction increases eNOS-Thr^495^ phosphorylation independently of the usual relaxation mechanisms. Previous studies have shown that eNOS-Thr^495^ phosphorylation is mainly regulated by three kinases, protein kinase C (PKC), Rho kinase (ROCK), and AMP-activated protein kinase (AMPK) (Fleming and Busse, 2003; Heiss and Dirsch, 2014). Among the tested inhibitors specific for each kinase, bisindolylmaleimide I (a selective PKC inhibitor) clearly inhibited the ASPD-induced increase in eNOS-P-Thr^495^, but Y-27632 (a ROCK inhibitor) and compound C (an AMPK inhibitor) did not (Figure 6A). To further confirm the involvement of PKC, we used another inhibitor that works differently: While bisindolylmaleimide I competes at ATP binding site of PKC, calphostin C inhibits the interaction between diacylglycerol and the PKC-regulatory domain (Iida et al., 1989; Kobayashi et al., 1989; Toullec et al., 1991). As shown in Figure 6B, calphostin C completely inhibited the ASPD-induced increase in eNOS-P-Thr^495^. Taken together these results show that PKC is a major regulator for the ASPD-induced increase in eNOS-Thr^495^ phosphorylation.

We next examined whether only ASPD have this effect, and not freshly dissolved Aβ_1-42_ on its own, which contains monomers, dimers, and low-molecular-weight oligomers (Jana et al., 2016; Ohnishi et al., 2015; Pryor et al., 2012). When human brain endothelial cells were treated for 6 hr with 1 μM freshly dissolved Aβ_1-42_ solution, the inactive form of eNOS increased as indicated by an increase of the eNOS-P-Thr^495^ ratio (Figure 6C left), as reported previously (Gentile et al., 2004). However, unlike ASPD, freshly dissolved Aβ_1-42_ solution did not induce PKC activation as shown clearly as shown clearly by the fact that there is in no change in the PKC-P-Ser^660^ ratio (Figure 6C right). Note that ASPD concentrationis calculated by using the average ASPD mass, 128 kDa (i.e., ∼30-mer Aβ assemblies). Therefore, 32 nM ASPD (used in Figure 6A) corresponds to 0.9 μM Aβ_1-42_. The above results indicated that only ASPD induce eNOS inactivation through PKC activation.

The next question is how the ASPD-NAKα3 interaction leads to PKC activation in the endothelial cells. We elucidated three possible activation mechanisms by using inhibitors specific for each mechanism: Tempol (a scavenger of ROS), BAPTA-AM (a chelator of intracellular calcium), or U-73122 (an inhibitor of phospholipase C (PLC)). Because all the tested activation mechanisms are known to be associated with auto-phosphorylation at Ser^660^ in PKC (Cosentino-Gomes et al., 2012; Feng and Hannun, 1998), the PKC activation was monitored by the ratio of PKC-Ser^660^ phosphorylation (PKC-P-Ser^660^). As shown in Figure 6D only tempol abolished the increase in PKC-P-Ser^660^ associated with PKC activation. Tempol also blocked the increase in eNOS-P-Thr^495^ induced by the ASPD-NAKα3 interaction (Figure 6E). These results consistently show that the ASPD-NAKα3 interaction induces PKC activation through ROS production.

The next question is where this ROS production occurs (Santilli et al., 2015), which we examined by using an ROS indicator, CellROX (Thermo Fisher Scientific, Waltham, MA). As shown in Figure 6F, CellROX detected an increase in ROS in the cytoplasm of endothelial cells treated with 35 nM ASPD for 6 hr. This increase was wiped out by pretreating the cells with inhibitors of mitochondrial ROS generation, YCG-063 or mito-tempol, but was not affected by pretreatment with NADPH oxidase inhibitors, VAS2870 or apocynin (Figure 6F). Although vascular ROS is also produced by xanthine oxidase (Santilli et al., 2015), the results in Figure 6F supported that mitochondria are the major source of the ROS production induced by the ASPD-NAKα3 interaction.

### Patient-derived ASPD increase eNOS-Thr^495^ phosphorylation through a mitochondrial ROS-PKC pathway

**Figure 7:**
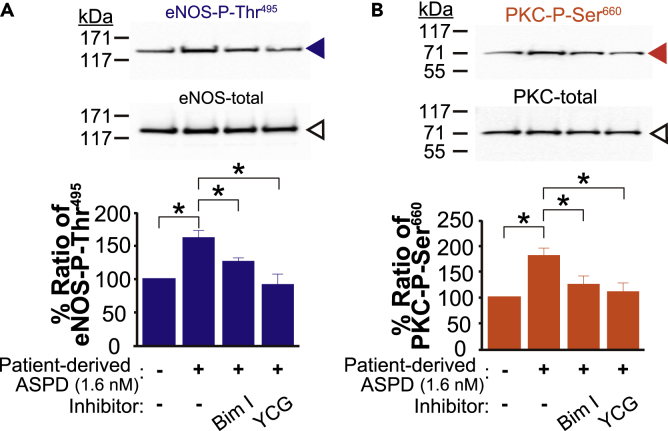
Patient-derived ASPD increase eNOS-Thr^495^ phosphorylation through a mitochondrial ROS-PKC pathway (A and B) Patient-derived ASPD were isolated from postmortem brains of the three AD patients according to the method established previously (Noguchi et al., 2009; Ohnishi et al., 2015) (see “method details”). Primary human endothelial cells were pretreated with bisindolylmaleimide I (Bim I, 5 μM) or YCG-063 (YCG, 50 μM) for 30 min, and were further treated with patient-derived ASPD (1.6 nM) for 6 hr (see “method details”). The ratios of eNOS-P-Thr^495^ to eNOS-total in A and PKC-P-Ser^660^ to PKC-total in B were determined as in Figure 6C (n = 4). Data are presented as means ± S.E. ∗*P* < 0.05 (ANOVA with Scheffé’s method).

**Figure 8:**
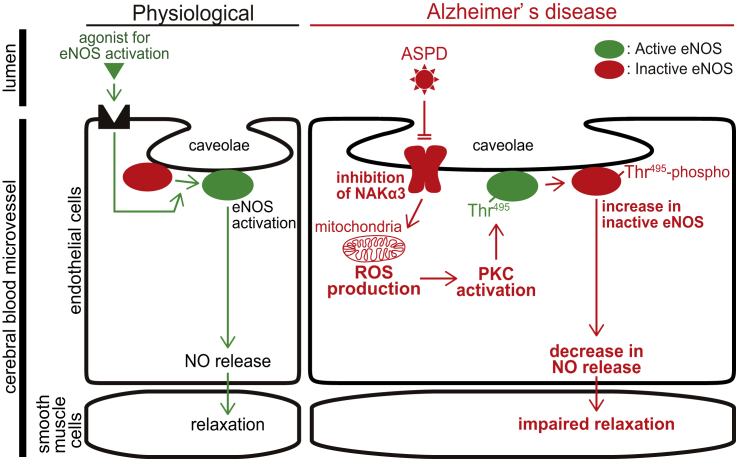
Schematic illustration of the mechanism of ASPD-induced suppression of eNOS activity in brain microvessel endothelial cells ASPD bind to cell-surface NAKα3 in caveolae on cerebral microvessel endothelial cells, promote mitochondrial ROS production, activate PKC, increase eNOS-Thr^495^ phosphorylation, and attenuate NO release, resulting in suppression of blood microvessel relaxation response.

Up to this section, because of the limited availability of patient-derived ASPD, we employed *in vitro*-reconstituted synthetic ASPD, which share the essential characteristics of patient-derived ASPD, including NAKα3 binding (Noguchi et al., 2009; Ohnishi et al., 2015), to prove that ASPD-NAKα3 interaction drives eNOS inactivation through mitochondrial ROS production and PKC activation. Finally, to validate this finding we used ASPD derived from the brains of the three AD patients displaying the most severe AD pathology and the highest ASPD concentrations, which we also used in our previous studies (Noguchi et al., 2009; Ohnishi et al., 2015), to confirm that patient-derived ASPD truly inactivate eNOS activity by increasing the phosphorylation of eNOS-Thr^495^ through a mitochondrial ROS-PKC pathway. Western blotting in Figure 7A showed that treatment of primary human endothelial cells with patient-derived ASPD for 6 hr did increase the eNOS-P-Thr^495^ ratio, and this increase was almost completely inhibited by treatment with either a PKC inhibitor (bisindolylmaleimide I) or a mitochondrial ROS inhibitor (YCG-063). Importantly, in association with the increase in the eNOS-P-Thr^495^ ratio, patient-derived ASPD increased the active form of PKC, as revealed by the increase in the PKC-P-Ser^660^ ratio, and this increase in active PKC was also blocked by treatment with bisindolylmaleimide I or YCG-063 (Figure 7B). These results support that both patient-derived and synthetic ASPD commonly drive eNOS inactivation through a mitochondrial ROS-PKC activation pathway, independently of the physiological relaxation system, in the cerebrovascular microvessels. We summarized our new findings obtained in this work as a scheme in Figure 8.

## Discussion

A decrease in eNOS activity leads to cerebrovascular dysfunctions such as reduced cerebral blood flow (Zhu et al., 2016). We found that the aberrant ASPD-NAKα3 interaction in cerebrovascular endothelial cells inactivates eNOS through a new system mediated by mitochondrial ROS production, independently of the physiological relaxation system (Figure 8). The current study opens up a new possibility that ASPD may contribute not only to neurodegeneration in the brain (Komura et al., 2019; Noguchi et al., 2009; Ohnishi et al., 2015), but also to cerebrovascular changes. This is in line with recent observations that vascular changes in the brain contribute to worsening of AD (Govindpani et al., 2019). Such a decrease of cerebral blood flow is responsible for alternating the physiological neurochemical environment, promoting the development of AD-related neuropathology, such as dysfunction and loss of neurons (Zlokovic, 2005). It is noteworthy that ASPD share the same toxic target, NAKα3, in neurons and in cerebral blood microvessels. Therefore, the ASPD-NAKα3 interaction may be a useful target for AD therapy.

The presence of NAKα3 on endothelial cells has not been well investigated, but our immunostaining data revealed the presence of NAKα3 as clusters approximately 230 nm in diameter on the surface of brain microvessel-derived endothelial cells (Figure 3A). This result suggests that NAKα3 may exist in a specific subdomain of the plasma membrane of the brain microvessel-derived endothelial cells, such as membrane microdomains (Leo et al., 2020), to which ASPD bind. Consistent with this observation, immunocytochemical studies showed that NAKα3 is present mainly in eNOS-existing caveolin-1-positive caveolae, but not in flotillin-1-positive lipid rafts (Figure 5A). The current result is also consistent with past studies showing that NAKα1 binds directly to caveolin-1 (Cai et al., 2008; Yosef et al., 2016). Notably, the proportion of eNOS coexisting with NAKα3 did not change significantly up to 1 hr after ASPD treatment (Figure 5A). Therefore, it seems that at least during the initial 1 hr ASPD do not change the distribution of NAKα3 on the cell membrane. The co-presence of NAKα3 and eNOS in caveolae may play a role in the physiology of endothelial cells by providing a platform for the recruitment and regulation of the signaling proteins involved in the NO-mediated signals, clarification of which need to await future studies. Our results collectively demonstrate that ASPD interact with NAKα3 in caveolae where eNOS is present in cerebrovascular endothelial cells.

The treatment of rat endothelial cells with a low concentration of ouabain was reported to increase intracellular calcium (Dong et al., 2004; Noel et al., 1990). We therefore expected that ASPD-induced PKC activation would occur in an intracellular calcium-dependent manner. However, the intracellular calcium chelator, BAPTA-AM, did not affect PKC activation (Figure 6D), suggesting that the ASPD-NAKα3 interaction activates PKC through a different pathway. Eventually, we proved that ROS mediates PKC activation by ASPD (Figure 6D), which was completely abolished by a selective scavenger of mitochondrial ROS production (Figure 6F). The molecular link between NAKα3 inactivation and mitochondrial ROS production remain to be clarified. Interestingly, mitochondrial ROS production was reported to be suppressed by activation of the NAKα3 pump by an NAK-DR region-specific antibody (Yan et al., 2016). These findings together support that NAK activity negatively regulates mitochondrial ROS production.

Suppression of the relaxation response of the blood vessels after Aβ treatment was first reported about two decades ago using rat-derived aortic rings, raising the possibility that Aβ may directly or indirectly reduce NO release (Crawford et al., 1998; Thomas et al., 1996). Since then, four papers have shown that the eNOS activity is actually decreased after the treatment of blood endothelial cells derived from umbilical vein, aorta, or basilar artery with high concentrations of Aβ (more than 5 μM) (Chisari et al., 2010; Gentile et al., 2004; Lamoke et al., 2015; Suhara et al., 2003). These previous studies suggested a possible link between Aβ and eNOS activity regulation, but it has been unknow whether Aβ directly causes the decrease of eNOS activity of the brain microvessels. Moreover, if Aβ does act directly on the eNOS activity of the brain microvessels, there still remained several questions that need to be clarified, such as what molecular entity of Aβ (Aβ monomers or a certain form of Aβ assemblies) actually works, through which target on the blood vessel Aβ acts, and what molecular mechanism leads to the decrease of eNOS activity. Here, we address these questions using primary human endothelial cells derived from the brain microvessels. We previously demonstrated that ∼30-mer Aβ assemblies, ASPD, are present in AD brains (Hoshi et al., 2003; Noguchi et al., 2009). ASPD bind to NAKα3 on the endothelial surface, leading to inhibition of eNOS activity by increasing an inactivated state of eNOS phosphorylated at Thr^495^ (Figures 3, 4, and 5). Thus, by showing that ASPD-specific antibody completely blocked reduction of the eNOS activity observed after ASPD treatment (Figure 4C), we showed that ASPD directly decreases the eNOS activity by binding to NAKα3. Importantly, while the previous four studies mentioned above observed a decrease in the eNOS-Ser^1177^ phosphorylation after Aβ treatment, which is a part of the physiological pathway for regulating eNOS activity, such a decrease in eNOS-Ser^1177^ phosphorylation was not detected in the case of ASPD (Figures 5C and 5D). Instead, as described above, we found that ASPD activate PKC, which increases eNOS-Thr^495^ phosphorylation, through mitochondrial ROS production (Figure 6). PKC has been reported to decrease eNOS-Ser^1177^ phosphorylation by activating Ser/Thr protein phosphatase 2A (PP2A) (Michell et al., 2001). Nevertheless, ASPD appeared not to significantly affect the level of eNOS-Ser^1177^ phosphorylation (Figures 5C and 5D). Thus, ASPD is likely to regulate the contraction of blood vessels in a way different from the physiological pathway for NO release which was previously reported (Michell et al., 2001).

To further clarify the molecular link between cerebrovascular dysfunction and parenchymal neuronal damage in the onset of AD induced by Aβ assemblies, one of the essential questions sequestered in the future is to understand the source of the Aβ assemblies in the blood microvessels. In the case of ASPD, we have already shown that ASPD are selectively formed in excitatory neurons and secreted (Komura et al., 2019). Therefore, it is natural to consider that ASPD are delivered to the cerebral blood vessels through apolipoprotein E (ApoE), clusterin, or brain meningeal lymphatics, as reported previously (Beeg et al., 2016, da Mesquita et al., 2018; Garai et al., 2014; Nelson et al., 2017). However, other possibilities, e.g., formation of ASPD in the cerebral blood microvessels, cannot be excluded, because Aβ precursor protein (APP) is also expressed on the surface of cerebral endothelial cells, even though the APP isoform profiles in endothelial cells are different from those in neurons (Grinberg et al., 2012; Kakuda et al., 2017; Kitazume et al., 2010). Even though in non-neuronal cells APP is mainly processed through the non-amyloidogenic pathway under physiological conditions (Kitazume et al., 2012), APP has been reported to be actually processed through the amyloidogenic pathway in endothelial cells (Kitazume et al., 2010) and in vascular smooth muscle cells obtained from tg2576 mouse lines expressing human Swedish APP, leading to formation of Aβ oligomers (Frackowiak et al., 2003, 2009). These findings are consistent with the possible formation of ASPD in the cerebral blood microvessels. Notably, if ASPD are truly formed in cerebral blood microvessels, as they are in neurons, we consider that a pathological trigger leading to ASPD formation in endothelial cells should be different from that in neurons owing to the differences in APP isoforms and APP processing between neurons and endothelial cells (Grinberg et al., 2012; Kakuda et al., 2017; Lasiecka and Winckler, 2011). We believe that further studies to identify the origin of cerebral vascular ASPD will not only deepen our understanding of ASPD themselves, but also help to understand the origin of other Aβ assemblies that accumulate in cerebral blood microvessels.

Interestingly, cerebrovascular ROS was reported to decrease cerebral blood flow through pericyte constriction of cerebral blood vessels (Nortley et al., 2019). Here, we found that ASPD induce mitochondrial ROS production in cerebral endothelial cells, but because released ROS could diffuse to affect nearby cells, it seems plausible that ASPD-induced ROS production in endothelial cells would also affect neighboring pericytes and block the physiological relaxation of cerebral blood vessels.

In this work, vascular function was examined using the aorta. Extending this work to monitor the microvascular blood flow in the brain might be possible using techniques such as laser speckle contrast imaging, but extensive optimization of the technology in terms of velocity distribution, effect of static scatterers, optimal speckle size, light penetration angle, contrast computation algorithms, etc. (Ayata et al., 2004; Bahadori et al., 2017) will be needed before this becomes feasible. Even though large blood vessels (such as the aorta) and brain microvessels use different types of vascular relaxation factors, i.e., NO for large vessels and endothelial-derived polarization factors (EDPH) such as H_2_O_2_ for microvessels, recent studies have shown that the production of H_2_O_2_ as an EDPH leading to vascular relaxation involves the conversion of superoxide produced by eNOS to H_2_O_2_ by endothelial Cu and Zn-superoxide dismutase (Shimokawa and Godo, 2016). Therefore, eNOS appears to play an important role in maintaining functional homeostasis in both types of vessels. Further studies are planned to examine how ASPD affect the brain microvessel systems including pericytes.

Decreased cerebral blood flow is an apparent risk factor for AD development (Zlokovic, 2005). Blocking the cerebrovascular toxicity of ASPD is thus expected to be an effective target to prevent worsening of AD. We have previously found an ASPD-binding tetrapeptide that is similar to the ASPD-binding domain on NAKα3, and surprisingly found that the treatment of ASPD with this peptide completely abolished ASPD-induced neuronal apoptosis by blocking the interaction of ASPD with NAKα3 (Ohnishi et al., 2015). Therefore, we expect that this may lead to a new AD therapeutic strategy based on dual attenuation of both the vascular and neuronal toxicities of ASPD.

### Limitations of the study

As already noted, we confirmed that ASPD did not affect the papaverine-induced relaxation response of isolated aortic rings. Accordingly, we concluded that binding of ASPD to endothelial cells was responsible for the effects shown in Figure 2. However, the above observation does not necessarily exclude the possibility that ASPD may also affect vascular smooth muscle cells, as has been reported for other types of Aβ (Hald et al., 2016), because aortic rings retain intact layer structures, so it is possible that ASPD may not penetrate the endothelial cell layer to reach the muscle cell layer below. Further work is planned to investigate this possibility.

## STAR★Methods

### Key resources table

**Table:** 

REAGENT or RESOURCE	SOURCE	IDENTIFIER
**Antibodies**		

Rabbit polyclonal ASPD-specific rpASD1 antibody	Noguchi et al., 2009	In house prepared
Mouse monoclonal ASPD-specific mASD3 antibody	Noguchi et al., 2009	In house prepared
Rabbit anti-Aβ_1-42_ antibody	Immuno-Biological Laboratories	Cat# 18582; RRID: AB_2341375
Rabbit anti-Aβ_1-40_ antibody	Immuno-Biological Laboratories	Cat# 18580; RRID: AB_2341496
Rat anti-CD34 antibody	BD Bioscience	Cat# 553731; RRID: AB_395015
Mouse monoclonal anti-α-actin antibody	Abcam	Cat# ab28052; RRID: AB_867491
Rabbit polyclonal anti-NAKα3 antibody	alomone labs	Cat# ANP-003; RRID: AB_2756681
Mouse monoclonal anti-von Willebrand Factor antibody	Santa Cruz Biotechnology	Cat# sc-365712; RRID: AB_10842026
Rabbit polyclonal anti-NAKα3 antibody	Santa Cruz Biotechnology	Cat# sc-16051-R; RRID: AB_2060974
Mouse anti-eNOS antiboy	BD Bioscience	Cat# 610297; RRID: AB_397691
Mouse monoclonal anti-flotillin-1 antibody	Santa Cruz Biotechnology	Cat# sc-74566; RRID: AB_2106563
Rabbit polyclonal anti-eNOS antibody	Santa Cruz Biotechnology	Cat# sc-654; RRID: AB_631423
Mouse anti-eNOS-P-Ser^1177^ antibody	BD Bioscience	Cat# 612393; RRID: AB_399751
Mouse anti-eNOS-P-Thr^495^ antibody	BD Bioscience	Cat# 612707; RRID: AB_399946
Mouse monoclonal anti-PKC antibody	Santa Cruz Biotechnology	Cat# sc-17769; RRID: AB_628139
Rabbit polyclonal anti-PKC-P-Ser^660^ antibody	Cell Signaling Technology Japan	Cat# 9371; RRID: AB_2168219
Goat polyclonal anti-NAKα3 antibody	Santa Cruz Biotechnology	Cat# sc-16052; RRID: AB_2227635
Mouse monoclonal anti-actin antibody	Merck-Millipore	Cat# MAB1501R; RRID: AB_2223041
Alexa Fluor 488-conjugated goat anti-mouse antibody	Molecular Probes	Cat# A11029; RRID: AB_138404
Alexa Fluor 488-conjugated goat anti-rabbit antibody	Molecular Probes	Cat# A11034; RRID: AB_2576217
Alexa Fluor 488-conjugated goat anti-rat antibody	Molecular Probes	Cat# A11006; RRID: AB_2534074
Alexa Fluor 568-conjugated goat anti-mouse antibody	Molecular Probes	Cat# A11031; RRID: AB_144696
Alexa Fluor 568-conjugated goat anti-rabbit antibody	Molecular Probes	Cat# A11011; RRID: AB_143157
HRP-conjugated goat anti-rabbit antibody	Thermo Fisher Scientific	Cat# 65-6120; RRID: AB_2533967
HRP-conjugated goat anti-mouse antibody	Thermo Fisher Scientific	Cat# A-10668; RRID: AB_2534058
Biotinylated donkey anti-goat antibody	Sigma-Aldrich Japan	Cat# SAB3700288

**Biological samples**		

Frontal cortex of AD patients’ brain	Brain bank of Brain Research Institute at Niigata University	https://www.bri.niigata-u.ac.jp
Patient-derived ASPD	Noguchi et al., 2009	In house prepared

**Chemicals, peptides, and recombinant proteins**		

Apocynin	Merck-Millipore	Cat# 178385
BAPTA-AM	Merck-Millipore	Cat# 196419
Bisindolylmaleimide I	Merck-Millipore	Cat# 203290
Calphostin C	Merck-Millipore	Cat# 208725
Compound C	Merck-Millipore	Cat# 171260
Mito-tempol	Cayman Chemical	Cat# 18796
Tempol	Sigma-Aldrich Japan	Cat# 176141
U-73122	Merck-Millipore	Cat# 662035
VAS2870	Merck-Millipore	Cat# 492000
Y-27632	Cayman Chemical	Cat# 10005583
YCG-063	Merck-Millipore	Cat# 557354
Avidin-peroxidase	Thermo Fisher Scientific	Cat# 21123
DAF-FM diacetate	Sekisui Medical	Cat# SKM423741
CellROX	Thermo Fisher Scientific	Cat# C10443
NuPAGE 3∼8% Tris-Acetate gels	Thermo Fisher Scientific	Cat# EA03752
NuPAGE Tris-Acetate SDS running buffer	Thermo Fisher Scientific	Cat# LA0041
HiMark protein standard	Thermo Fisher Scientific	Cat# LC5699
Lipofectamine 3000 reagent	Thermo Fisher Scientific	Cat# L3000001
Krebs-Henseleit solution	Sigma-Aldrich Japan	Cat# K3753
EGM-2MV medium	Lonza Japan	Cat# CC-3202
In-house synthesized Aβ_1-42_ peptides	Hoshi et al., 2003	In house prepared

**Critical commercial assays**		

Vectastain ABC kit	Vector Laboratories	Cat# PK-7200
BCA Protein Assay Kit	Thermo Fisher Scientific	Cat# 23225

**Experimental models: Cell lines**		

Human: Primary human endothelial cells derived from human brain microvessels	Cell Systems	Cat# ACBRI 376

**Experimental models: Organisms/strains**		

Rat: Slc:Wistar	Japan SLC	Cat# Slc:Wistar

**Oligonucleotides**		

Primer: *ATP1A3* forward: 5’-CGCCG GGACCTGGATGACCTC-3’	Fransen et al., 2001	N/A
Primer: *ATP1A3* reverse: 5’-CGGA TCACCAGGGCTTGCTGG -3’	Fransen et al., 2001	N/A
Primer: *GAPDH* forward: 5’-CAAGG TCATCCATGACAACTTTG-3’	Ouyang et al., 2014	N/A
Primer: *GAPDH* reverse: 5’-GTCC ACCACCCTGTTGCTGTAG-3’	Ouyang et al., 2014	N/A
siRNA: *ATP1A3* siRNA	Thermo Fisher Scientific	Cat# s1724
siRNA: MOCK siRNA	Thermo Fisher Scientific	Cat# 4390843

**Software and algorithms**		

Zen2009 software	Carl Zeiss	N/A
CQ1 software	Yokogawa Electric Corp	https://www.yokogawa.co.jp/library/documents-downloads/software/lsc-cq1-software/
Statcel2 software	OMS Publication	N/A

**Other**		

Light microscope AX80T	Olympus	N/A
Confocal laser-scanning microscope LSM710	Carl Zeiss	N/A
Force-displacement transducer AP-5	Medical Kishimoto	N/A
Confocal quantitative imaging cytometer CQ1	Yokogawa Electric Corp	N/A

### Resource availability

#### Lead contact

Further information and requests for resources should be directed to and will be fulfilled by the Lead Contact, Minako Hoshi (minako.stella.hoshi.37@fbri.org).

#### Materials availability

This study did not generate new unique reagents.

### Experimental model and subject details

#### Wistar rat

The Animal Care and Experimentation Committees of Kobe Gakuin University, Foundation for Biomedical Research and Innovation at Kobe, and TAO Health Life Pharma Co., Ltd approved the experiments using animals. Seven-week-old male Wistar rats were purchased from Japan SLC (Slc:Wistar; Japan SLC, Shizuoka, Japan). The rats were housed at no more than 4 animals per cage under 12-hr light and 12-hr dark cycles at a room temperature of 25°C and had free access to food and water. All animals had been properly quarantined according to the monitoring reports of Japan SLC.

#### Primary human endothelial cells derived from human brain microvessels

Primary human brain microvascular endothelial cells (ACBRI376) isolated from normal healthy donor tissues were obtained from Cell Systems (Kirkland, WA) at passage 3. Details of the donors (including gender) are not available. The endothelial cells were cultured on collagen I-coated dishes and glass plates with EGM-2MV medium (CC-3202; Lonza Japan, Tokyo, Japan) at 37°C under 5% CO_2_, according to the manufacturer’s protocol. The endothelial cells at passage at 6–8 were utilized for the study. Because the cells were guaranteed to conform to the description specified in the Cell Systems Certificate, we did not authenticate these cells in our laboratory.

### Method details

#### Immunohistochemical staining of AD brains

The Bioethics Committees and the Biosafety Committees of Niigata University, Kyoto University, Foundation for Biomedical Research and Innovation at Kobe, and TAO Health Life Pharma Co., Ltd. approved the experiments using human materials.

Immunohistochemical DAB staining of brain sections from the three AD patients who showed the most severe AD pathologies and the highest ASPD levels (see Table 1 for patient profiles) (Noguchi et al., 2009) was performed as previously described (Noguchi et al., 2009). Briefly, paraffin-embedded 4 μm serial sections of prefrontal cortex were prepared from post-mortem brains of the patients. For Aβ_1-40_ or Aβ_1-42_ staining, the sections were pretreated with 100% formic acid for 5 min at room temperature. In contrast, no pretreatment was generally performed for ASPD staining (Noguchi et al., 2009). The sections, with or without pretreatment, were treated with 0.3% (v/v) H_2_O_2_-methanol for 60 min, then incubated with PBS without calcium and magnesium (PBS, 27575, Nacalai tesque, Kyoto, Japan)/10% (v/v) normal goat serum (Immuno-Biological Laboratories, Gunma, Japan) for 30 min at room temperature, and further treated with a blocking kit (PK-7200, Vector Laboratories, Burlingame, CA) for 30 min. These sections were incubated overnight at 4°C with primary antibody against ASPD (ASPD-specific rpASD1 antibody (Noguchi et al., 2009), 1.25 μg/mL), Aβ_1-42_ (18,582, 1:200, Immuno-Biological Laboratories), or Aβ_1-40_ (18,580, 1:200, Immuno-Biological Laboratories) in the presence of 10% normal goat serum in PBS, followed by incubation with biotinylated secondary antibody (PK-7200, Vector Laboratories) for 60 min at room temperature. Immunoreactivities were detected by the avidin-biotin-peroxidase complex method using a Vectastain ABC kit (PK-7200, Vector Laboratories) and an ImmPACT DAB staining kit (SK-4105, Vector Laboratories). Counterstaining was carried out with Mayer’s hematoxylin. Images were captured by using a light microscope AX80T (Olympus, Tokyo, Japan) with a digital camera DP70 (Olympus).

Immunohistochemical fluorescence staining of the serial sections of the same AD brains were performed as previously described with some modifications (Komura et al., 2019) as shown below. For Aβ_1-40_ or Aβ_1-42_ staining, the sections were pretreated with formic acid for 5 min. In contrast, no pretreatment was generally performed for ASPD staining (Noguchi et al., 2009). The sections, with or without pretreatment, were incubated with PBS/10% (v/v) normal goat serum for 30 min at room temperature. These sections were incubated overnight at 4°C with primary antibody against ASPD (ASPD-specific mASD3 antibody (Noguchi et al., 2009), 0.5 μg/mL; or ASPD-specific rpASD1 antibody (Noguchi et al., 2009), 1.25 μg/mL), CD34 (553731, 1:100, BD Bioscience, Franklin Lakes, NJ), and α-actin (ab28052, 1:100, Abcam, Cambridge, UK) in the presence of PBS/10% (v/v) normal goat serum, and then incubated with the appropriate secondary antibodies (Alexa Fluor 488-conjugated goat anti-mouse antibody, A11029; Alexa Fluor 488-conjugated goat anti-rabbit antibody, A11034; Alexa Fluor 488-conjugated goat anti-rat antibody, A11006; Alexa Fluor 568-conjugated goat anti-mouse antibody, A11031; and Alexa Fluor 568-conjugated goat anti-rabbit antibody, A11011; 1:1000, Molecular Probes, Waltham, MA) for 1 hr at room temperature. These sections were mounted with ProLong Gold anti-fade reagent (P36934, Invitrogen, Waltham, MA). Fluorescence images were captured with a confocal laser-scanning microscope LSM710 (Carl Zeiss, Oberkochen, Germany) with a x100 oil-immersion objective lens.

#### Isolation of rat aortas

To perform the *ex vivo* relaxation experiments and the immunohistochemical staining of rat blood vessels, the rats were sacrificed by bleeding from the carotid arteries under isoflurane anesthesia. Aortic rings were cut out and the attached adipose tissue was immediately removed in Krebs-Henseleit solution (118.4 mM NaC1, 4.7 mM KC1, 2.5 mM CaCl_2_, 1.2 mM KH_2_PO_4_, 1.2 mM MgSO_4_, 25.0 mM NaHCO_3_, and 11.1 mM glucose, 37°C) (K3753; Sigma-Aldrich Japan, Tokyo, Japan).

#### *Ex vivo* relaxation of rat blood vessels

The *ex vivo* vascular study was performed as previously described with some modifications (Sasahara et al., 2013), as shown below. The aortas prepared (see “Isolation of rat aortas”) were cut into 3 mm vessel rings and randomly divided into each experimental group. These vessel rings were vertically fixed under a preload of 1.0 g in an organ bath filled with Krebs-Henseleit solution continuously aerated with 95% O_2_/5% CO_2_ gas, and allowed to equilibrate for 60 min. The vessel rings were treated with ASPD (46 nM) or ouabain (100 nM) for 60 min. In the case of ASPD preincubated with ASPD-specific mASD3 antibody, ASPD (920 nM) were preincubated with mASD3 antibody (0.1 mg/mL) for 2 hr at 4°C without agitation, as previously reported (Ohnishi et al., 2015), and the vessel rings were treated with the mASD3 antibody-preincubated ASPD (46 nM) for 60 min. The treated vessel rings were initially constricted by phenylephrine (1 μM) treatment for no more than 10 min. Then, the relaxation response was initiated by the addition of carbachol (0.001–100 μM), which usually reached the maximum level within 2–3 min. The relaxation was calculated as described below. After the carbachol-induced relaxation process was saturated, the maximal relaxation response of the vessel rings, evoked by papaverine (0.1 mM), was further measured. The isometric tension change of vessel rings was monitored with a force-displacement transducer (AP-5; Medical Kishimoto, Kyoto, Japan) coupled to a chart recorder (SS-250 F; SEKONIC, Tokyo, Japan), according to the manufacturer’s protocol. Relaxation response data are expressed as a percentage to the phenylephrine-induced constriction response.

#### Immunohistochemical staining of rat aortas

Immunohistochemical fluorescence staining of the sections prepared from rat aortas was performed essentially as described above (see “Immunohistochemical analyses of AD patient brains”) with some modifications. The aortas isolated from rat (see “Isolation of rat aortas”) were fixed with 4% (w/v) paraformaldehyde (PFA) overnight and were embedded in paraffin wax, and 4 μm sections of rat aortas were prepared. To stain NAKα3, the sections were incubated with 10 mM citrate buffer for 30 min at 95°C. After the incubation, the sections were further incubated with primary antibody against NAKα3 (ANP-003, 1:200; Alomone Labs, Jerusalem, Israel) and von Willebrand factor glycoprotein (sc-365712, 1:50; Santa Cruz Biotechnology, Dallas, TX), and then incubated with the appropriate secondary antibodies (see above). These sections were mounted with ProLong Gold anti-fade reagent (P36934, Invitrogen). Fluorescence images were captured with LSM710 with a x100 oil-immersion objective lens.

#### Immunocytochemical staining

Primary endothelial cells were propagated until the density reached ∼80%, and the culture medium was replaced. Twenty-four hours later, the cells were treated with ASPD at the indicated concentration for 10 min or 60 min. Then, the cells were washed twice with PBS and fixed with 4% (w/v) PFA for 20 min at 37°C. To prevent delocalization of proteins, staining was performed immediately. The fixed cells were treated with 2 mg/mL glycine for 10 min at room temperature, permeabilized with 0.2% (v/v) Triton X-100 for 5 min at room temperature, and pretreated with PBS/3% (w/v) bovine serum albumin (A7030, Sigma-Aldrich Japan)/10% (v/v) normal goat serum for 30 min at room temperature. These cells were incubated overnight with primary antibody against ASPD (ASPD-specific mASD3 antibody (Noguchi et al., 2009), 0.5 μg/mL; or ASPD-specific rpASD1 antibody (Noguchi et al., 2009), 1.25 μg/mL), NAKα3 (sc-16051-R, 0.4 μg/mL, Santa Cruz Biotechnology), eNOS (610297, 1:200, BD Bioscience), and flotillin-1 (sc-74566, 1:100; Santa Cruz Biotechnology) at 4°C, and incubated with the appropriate secondary antibodies (Alexa Fluor 488-conjugated goat anti-rabbit antibody, A11034; and Alexa Fluor 568-conjugated goat anti-mouse antibody, A11031, 1:1000, Molecular Probes) with counterstaining by 4′,6-diamidino-2-phenylindole (DAPI, 1:500, Dojindo Molecular Technologies, Kumamoto, Japan) for 60 min at room temperature. These cells were mounted with ProLong Gold anti-fade reagent. Fluorescence images were captured with LSM710 (Carl Zeiss) with a x100 oil-immersion objective lens, and z-stacked images were taken at 2 μm intervals. The line scan analysis of fluorescence intensities was performed with a Zen 2009 software (Carl Zeiss) using the 2D images captured. The vertical section image was prepared from the z-stacked images with Zen 2009 software. The weighted colocalization coefficients was analyzed using Zen 2009 software (Zeiss) as previously described (Komura et al., 2019). Briefly, the weighted colocalization coefficients represent the number of red (or green) pixels that colocalize with green (or red) pixels divided by the total number of red (or green) pixels. To quantitate the Mander correlation coefficient, approximately 500 cells in 5 view fields/well were acquired with a confocal quantitative imaging cytometer CQ1 (Yokogawa Electric Corp., Tokyo, Japan), and total NAKα3 staining and ASPD-bound NAKα3 staining were analyzed with CQ1 software. CQ1 software quantified only the NAKα3 staining on the endothelial cells, excluding staining derived from antibodies non-specifically bound to the culture dish bottom and non-specific staining in the nucleus. Note that the anti-NAKα3 antibody selectively reacts with NAKα3, except for the signals around nucleoli (in the case of non-neuronal cells, including endothelial cells, the anti-NAKα3 antibody shows thick and aggregated signals in nuclei due to non-specific binding (see Figure 3E in (Ohnishi et al., 2015)).

#### Aβ_1-42_ synthesis

To prepare synthetic ASPD, highly soluble Aβ_1-42_ peptide was synthesized in-house, as previously reported (Hoshi et al., 2003; Komura et al., 2019; Noguchi et al., 2009; Ohnishi et al., 2015). Aβ_1–42_ peptide was synthesized on an Applied Biosystems model 433A peptide synthesizer using solid-phase N-(9-fluorenyl) methoxycarbonyl (Fmoc) chemistry on Fmoc-Ala-NovaSyn-TGA resin (0.19 mmol/g; Novabiochem) (>75% yield). The synthesized peptide was cleaved and deprotected in a solution containing phenol (0.15 g), trifluoroacetic acid (TFA, 1.65 mL), Milli-Q water (0.05 mL), thioanisole (0.1 mL), and ethanedithiol (0.05 mL) (2 mL/200 mg resin). Crude peptides were precipitated by adding 30 mL of ice-cold diethyl ether and the precipitates were washed twice, air-dried for 20 min, further dried in a vacuum for 1 hr, then completely dissolved in a solution containing 0.1% (v/v) TFA and 30% (v/v) acetonitrile (ACN) on ice, and lyophilized. The Aβ_1–42_ peptide was purified by ZORBAX 300 Extend-C18 (21.2 mm × 250 mm, 5 μm; Agilent) reverse-phase chromatography with linear gradient elution (8–32% (v/v) ACN in 70 mM NH_4_OH). The purified peptides were immediately lyophilized, redissolved in a solution containing 0.1% (v/v) TFA and 30% (v/v) ACN on ice (∼150 μM), lyophilized again, and kept at −30°C until used. On average, 40–80 mg of purified Aβ_1–42_ peptide was obtained in the 0.1-mmol scale synthesis. Purity was confirmed by analytical HPLC, quantitative amino acid analysis, and MALDI-TOF/MS.

Before ASPD preparation, Aβ_1–42_ was completely dissolved in 1,1,1,3,3,3-hexafluoro-2-propanol (HFIP for HPLC; Kanto Chemical Co.) at 80–100 μM by incubating the peptide solution overnight at 4°C and for another 3 hr at 37°C, and finally lyophilized (∼40 nmol per tube). The Aβ_1–42_ concentration in this step must be kept below 100 μM to maintain the monomeric state. This step was repeated three times. The lyophilized peptide was kept at −30°C. We had long used HFIP from Sigma-Aldrich for lyophilization, but we recently found that this usually contained ∼1.3 mM bis(2-ethylhexyl) phthalate (DEHP). This means that solutions of peptide lyophilized in Sigma-Aldrich HFIP usually contained ∼0.65 mM DEHP when the final peptide concentration was 50 μM. Therefore, when we used HFIP in which DEHP was undetectable, we added DEHP (0.65 mM final concentration; Tokyo Chemical Industry Co.) when the lyophilized peptide was initially dissolved in anhydrous dimethyl sulfoxide (Sigma-Aldrich) to ensure consistency with our previous conditions (Hoshi et al., 2003; Matsumura et al., 2011; Noguchi et al., 2009).

#### ASPD preparation

ASPD are neurotoxic, spherical Aβ assemblies of 10–15 nm in diameter (measured by TEM) that are recognized by ASPD-specific antibodies (Matsumura et al., 2011; Noguchi et al., 2009). Synthetic ASPD were prepared in the same manner in (Noguchi et al., 2009), as briefly shown below. Using in-house-prepared highly soluble Aβ_1-42_ peptides (essential for obtaining ASPD; see “Aβ synthesis”), synthetic ASPD were formed in 50 μM Aβ_1-42_ solution in F12 buffer without L-glutamine and phenol red by slowly rotating the solution for 16 hr at 4°C. The level of ASPD to Aβ_1-42_ in this Aβ_1–42_ solution after slow rotation is usually ∼30%. Synthetic ASPD were obtained in the fraction that passed through 0.22-μm filters, but was retained on 100-kDa MWCO filters (Sartorius Japan, Tokyo, Japan) according to the manufacturer’s protocol. Synthetic ASPD quality was confirmed by dot blotting, TEM, and amino acid analysis (Matsumura et al., 2011).

The Bioethics Committees and the Biosafety Committees of Niigata University, Kyoto University, Foundation for Biomedical Research and Innovation at Kobe, and TAO Health Life Pharma Co., Ltd. approved the experiments. Patient-derived ASPD were prepared from soluble extracts of AD brains, using our established method (Noguchi et al., 2009). Briefly, freshly frozen blocks from autopsied AD brains were homogenized to 0.15 g/mL in ice-cold F12 buffer without L-glutamine and phenol red containing 1 mM EDTA, 1 mg/mL pepstatin, and complete protease inhibitor cocktail (Sigma-Aldrich Japan) using a Potter Teflon/glass homogenizer (10 strokes at 1,200 rpm). The initial supernatant was collected as C1S following centrifugation at 104,300 g (TLA100.4) at 4 °C for 1 hr. The pellet was further homogenized and the second supernatant, which contained patient-derived ASPD, was collected as C2S following centrifugation for 1 hr at 104,300 g (TLA100.4) at 4°C. In order to obtain large amounts of starting materials, soluble C2S fractions were repeatedly extracted (up to 9 times; C2S_1-9_) as described above. Protein concentrations were determined by Bradford protein assay (29,449, Nacalai tesque) using an IgG standard (500-0005, Bio-Rad). Then, to exclude low-molecular-weight oligomers, the mixed supernatants C2S_4-9_ were filtered with 100-kDa MWCO filter (Amicon Ultra UFC210024, Merck-Millipore, Burlington, MA), according to the manufacturer’s protocol. Patient-derived ASPD were concentrated in the retentate fraction of the 100-kDa MWCO filters. In order to obtain patient-derived ASPD, 20 mL of the supernatant C2S_4-9_ was concentrated to 200 μL. The quality of patient-derived ASPD was confirmed by dot blotting (Matsumura et al., 2011).

#### Freshly dissolved Aβ_1-42_ solution

Freshly dissolved Aβ_1-42_ solution (50 μM) was prepared by dissolving in-house-prepared highly soluble Aβ_1-42_ peptides freshly in F12 buffer without L-glutamine and phenol red just before use. The solution was used immediately.

#### Western blotting

Primary endothelial cells were propagated until the density reached ∼80%, and the culture medium was replaced. Twenty-four hours later, in the case of inhibitor pretreatment, the cells were pretreated with bisindolylmaleimide I (5 μM; 203290, Merck-Millipore), Y-27632 (10 μM; 10005583; Cayman Chemical, Ann Arbor, MI), compound C (10 μM; 171260, Merck-Millipore), calphostin C (0.3 μM; 208725, Merck-Millipore), tempol (3 mM; 176141, Sigma-Aldrich Japan), BAPTA-AM (30 μM; 196419, Merck-Millipore), U-73122 (10 μM; 662035, Merck-Millipore), or YCG-063 (50 μM; 557354, Merck-Millipore) for 30 min. Calphostin C-treated cells were illuminated by light for the first 15 min of 30 min because calphostin C needs light exposure to activate (Iida et al., 1989; Kobayashi et al., 1989). The cells were then treated with synthetic or patient-derived ASPD at the indicated concentration, or with freshly dissolved Aβ_1-42_ (1 μM), for 6 hr, except for the western blotting to determine the level of eNOS-Ser^1177^ phosphorylation, which required another 5-min treatment of the cells with carbachol (1 μM). Then, the cells were washed twice with PBS and the whole-cell protein was extracted with RIPA buffer (107 mM NaC1, 50 mM Tris-HCl, 5 mM EDTA, 0.1% (w/v) SDS, 0.5% (w/v) sodium deoxycholate, 1% (v/v) NP-40, 1 μg/mL pepstatin, cOmplete Mini (Sigma-Aldrich Japan), and PhosSTOP (Sigma-Aldrich Japan)). SDS-PAGE/Western blotting was performed as previously described with some modifications (Komura et al., 2019), as follows. The protein concentration of the RIPA lysates was quantified with a BCA Protein Assay Kit (23225, Thermo Fisher Scientific), according to the manufacturer’s protocol. Except for NAKα3 detection, 10 μg protein/lane was separated under denaturing conditions on 3–8% Tris-Acetate gels with Tris-Acetate SDS running buffer (NuPAGE, Thermo Fisher Scientific), along with HiMark protein standard (Thermo Fisher Scientific) as a protein marker. After transferring the proteins to 0.2 μm nitrocellulose membrane, the membrane was blocked with 5% (w/v) skim milk/0.05% (v/v) Tween 20 for 1 hr at room temperature, and was probed with a primary antibody against eNOS (sc-654, 1:200; Santa Cruz Biotechnology), phosphorylated eNOS (for phosphorylated Ser^1177^, 612393, 1:1000, BD Biosciences; for phosphorylated Thr^495^, 612707, 1:1000, BD Biosciences), PKC (sc-17769, 1:200, Santa Cruz Biotechnology), phosphorylated PKC-Ser^660^ (9371, 1:1000; Cell Signaling Technology Japan, Tokyo, Japan), or actin (MAB1501R, 1:500; Merck-Millipore) overnight at 4°C. The bands were detected by treating the membrane with the appropriate HRP-conjugated secondary antibody (goat anti-mouse antibody, A-10668, 1:5000, Thermo Fisher Scientific; goat anti-rabbit antibody, 65–6120, 1:10,000, Thermo Fisher Scientific) in 5% (w/v) skim milk/0.05% (v/v) Tween 20 for 1 hr at room temperature, followed by treatment with SuperSignal West Femto chemiluminescent substrates (Thermo Fisher Scientific), and were quantified using LAS-4000 Mini (GE Healthcare Japan, Tokyo, Japan). To increase the detection sensitivity of Western blotting for NAKα3 detection by anti-NAKα3 antibody (sc-16052, 1:250, Santa Cruz Biotechnology), we modified the above method in three points: First, we increased the applied protein amount (70 μg protein/lane); second, we used 3% (w/v) bovine serum albumin/20% (v/v) normal goat serum as a blocking solution; and third, we used biotinylated secondary antibody (SAB3700288, 1:1000, Sigma-Aldrich Japan) and avidin-peroxidase (21123, 1:3000, Thermo Fisher Scientific) for detection. Quantitative data are shown as the densitometric ratio of phosphorylated eNOS (eNOS-P-Ser^1177^ or eNOS-P-Thr^495^) to total eNOS, phosphorylated PKC (PKC-P-Ser^660^) to total PKC, or NAKα3 to actin.

#### Reverse transcription-PCR

The primary endothelial cells were propagated until the density reached ∼80%, and the culture medium was replaced. Twenty-four hours later, total RNA was extracted using TRIzol reagent (15596026, Thermo Fisher Scientific). RNA (0.5 μg) was reverse-transcribed using ReverTra Ace reaction mixture (FSQ-201, TOYOBO, Osaka, Japan) with oligo (dT) primer. The reaction mixtures were incubated at 42°C for 20 min, 99°C for 5 min, then 4°C for 5 min to synthesize the first strand of cDNA. The cDNA was then mixed with KOD FX PCR reaction mixture (KFX-101, TOYOBO) with forward and reverse primers for *ATP1A3* (forward primer 5′-CGCCGGGACCTGGATGACCTC-3′ and reverse primer 5′-CGGATCACCAGGGCTTGCTGG-3′; the PCR product is 434 bp (Fransen et al., 2001)) or those for *GAPDH* (forward primer 5′-CAAGGTCATCCATGACAACTTTG-3′ and reverse primer 5′-GTCCACCACCCTGTTGCTGTAG-3′; the PCR product is 496 bp (Ouyang et al., 2014)). PCR reaction was performed under the following conditions: Initial denaturation at 98°C for 2 min; 30 cycles of denaturation at 98°C for 10 s, annealing at 55°C for 30 s, and extension at 68°C for 30 s; final extension at 68°C for 7 min. PCR products were separated on 1.5% agarose gel and visualized using ethidium bromide.

#### Transfection of siRNA

*ATP1A3* siRNA (s1724, Thermo Fisher Scientific) was mixed with Lipofectamine 3000 reagent (L3000001, Thermo Fisher Scientific), according to the manufacturer’s protocol. As a negative control, a commercially available and widely accepted MOCK siRNA (4390843, Thermo Fisher Scientific) was utilized and prepared as described above. The primary endothelial cells propagated until the density reached ∼80% were treated with the siRNA mixture, and 6 hr later, the culture media containing siRNA mixture was replaced. Three days later, the cells were treated with ASPD at the indicated concentrations for 10 min (for immunocytochemical staining) or for 6 hr (for Western blotting). The cells for staining were fixed with 4% (w/v) PFA and stained as described above (see “immunocytochemical staining”). The whole-cell proteins were extracted, and Western blotting was performed as described above (see “western blotting”).

#### Measurement of NO release

The primary endothelial cells were propagated until the density reached ∼80%, and the culture medium was replaced. Twenty-four hours later, some cells were pretreated with ASPD (0.3, 3, or 32 nM) for 1, 3, or 6 hr. In the case of ASPD preincubated with ASPD-specific mASD3 antibody, ASPD (640 nM) were preincubated with mASD3 antibody (0.1 mg/mL) for 2 hr at 4°C without agitation, as previously reported (Ohnishi et al., 2015), and the cells were treated with the mASD3 antibody-preincubated ASPD (32 nM) for 3 hr. The cells were washed twice with HBSS containing Mg^+^ and Ca^2+^ (HBSS, 09735-75, Nacalai tesque) and were loaded with a fluorescent NO indicator DAF-FM diacetate (SKM423741, Sekisui Medical, Tokyo, Japan), according to the manufacturer’s protocol. The fluorescence intensity of DAF-FM loaded into the cells was measured before and after the carbachol (1 μM) treatment for 5 min (the fluorescence intensities before and after the carbachol treatment are defined as F_0_ and F_t_, respectively) using a CQ1 with a x20 objective lens, which acquired approximately 500 cells in 5 view fields per well. The sum of fluorescence intensity in each well was quantified using CQ1 software. As a negative control, we confirmed that pretreatment with NOS inhibitor *L*-NAME (40 μM; 80210, Cayman Chemical) for 30 min blocked the increase of fluorescence intensity by carbachol (1 μM) (∼2.01 ± 0.53%, n = 4, *P*< 0.001), and therefore the increase of DAF-FM fluorescence intensity reflects eNOS activity and consequently NO release in the endothelial cells. NO release data are expressed as a ratio of F_t_ to F_0_.

#### Measurement of ROS production

The primary endothelial cells were propagated until the density reached ∼80%, and the culture medium was replaced. Twenty-four hours later, in the case of inhibitor pretreatment, the cells were pretreated with YCG-063 (50 μM; 557354, Merck-Millipore), mito-tempol (100 μM; 18,796, Cayman Chemical), VAS2870 (10 μM; 492000, Merck-Millipore), or apocynin (20 μM; 178385, Merck-Millipore) for 30 min. All cells were then treated with ASPD (35 nM) for 6 hr, washed twice with HBSS, and were then loaded with a fluorescent ROS indicator CellROX (C10443, Thermo Fisher Scientific), according to the manufacturer’s protocol. The fluorescence intensity of CellROX loaded into the cells was measured using CQ1 with a x20 objective lens, which acquired approximately 500 cells in 5 view fields per well. The sum of fluorescence intensity in each well was quantified using CQ1 software. Data are show as a percentage set to 100 in the ASPD-treated group.

### Quantification and statistical analysis

All data are expressed as mean ± S.E. We used Statcel 2 software (OMS Publication, Tokyo, Japan) for statistical analyses. No data points were excluded from the analysis. Statistical comparisons were performed with the unpaired Welch's *t*-test between two groups or with one-way analysis of variance (ANOVA) followed by pair-wise comparisons using Scheffé’s method. Differences were considered significant at *P* < 0.05.

## Data Availability

• All data reported in this paper will be shared by the lead contact upon request. • This study did not generate datasets and did not report original code. • Any additional information required to reanalyze the data reported in this paper is available from the lead contact upon request.
