# Supplementary material for: Alzheimer's Aβ assembly binds sodium pump and blocks endothelial NOS activity via ROS-PKC pathway in brain vascular endothelial cells
Source: iScience. 2021 Aug 4;24(9):102936. doi: 10.1016/j.isci.2021.102936 (PMC8379508; doi:10.1016/j.isci.2021.102936)
Supplement: Document S1. Figure S1 [file mmc1.pdf]

iScience, Volume *24*

Supplemental information

Alzheimer's Aβ assembly binds sodium pump and blocks endothelial NOS activity via ROS-PKC pathway in brain vascular endothelial cells

Tomoya Sasahara, Kaori Satomura, Mari Tada, Akiyoshi Kakita, and Minako Hoshi

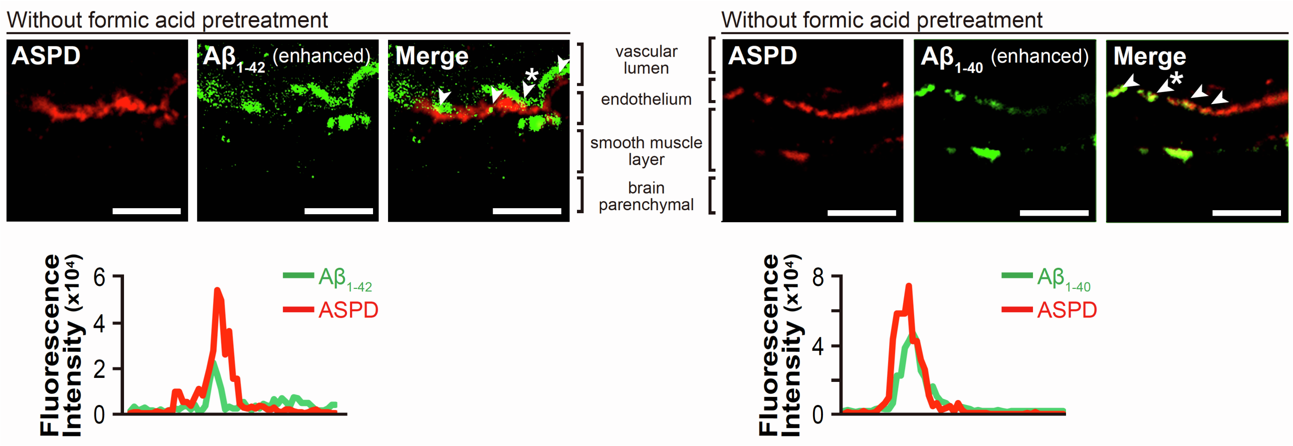

Figure S1 – Enhanced fluorescence image of Aβ staining (related to figure 1C).

The enhanced fluorescence images of $A\beta_{1-42}$ and $A\beta_{1-40}$ stainings without formic acid pretreatment in Fig. 1C are shown in the upper panels. White arrowheads in the upper panels indicate ASPD staining overlapped with Aβ staining. The line-scan analysis (indicated by white arrowhead with * in the upper panel) of the fluorescence intensity of $A\beta_{1-42}$ and $A\beta_{1-40}$ (green line), or ASPD (red line) was determined using Zen2009 software (see "METHOD DETAILS"). Scale bars: 5 μm.
